# Combination effect of optical defocus and low dose atropine in myopia control: Study protocol for a randomized clinical trial

**DOI:** 10.1371/journal.pone.0306050

**Published:** 2024-06-26

**Authors:** Rachel Ka Man Chun, Ying Hon, Tsz Kin Law, Kryshell Yu Qi Wong, Chi Ho To, Kendrick C. Shih, Christopher Kai Shun Leung, Dennis Yan Yin Tse

**Affiliations:** 1 School of Optometry, The Hong Kong Polytechnic University, Kowloon, Hong Kong; 2 Centre for Eye and Vision Research (CEVR), Shatin, Hong Kong; 3 Research Centre for SHARP Vision (RCSV), The Hong Kong Polytechnic University, Kowloon, Hong Kong; 4 School of Optometry & Vision Sciences, Cardiff University, Cardiff, United Kingdom; 5 Department of Ophthalmology, LKS Faculty of Medicine, The University of Hong Kong, Pok Fu Lam, Hong Kong; PLOS: Public Library of Science, UNITED KINGDOM

## Abstract

**Background:**

Myopia, characterized by excessive axial elongation of the eyeball, increases risks of having sight-threatening diseases and impose a financial burden to healthcare system. Although myopic control interventions showed their effectiveness in slowing progression, the efficacy varies between individuals and does not completely halt progression. The study aims to investigate the efficacy of combining 0.01% atropine administered twice daily with optical defocus for myopia control in schoolchildren.

**Methods and design:**

This is a prospective, parallel-group, single-blinded, randomized, active-control trial (ClinicalTrials.gov identifier: NCT06358755). Myopic schoolchildren with no previous myopic control interventions aged between 7 to 12 years will be recruited. They will be randomly allocated into two groups (n = 56 per group) after baseline measurement. Both groups will receive 0.01% atropine twice per day for 18 months (one drop in the morning and the other drop at night before bedtime). Defocus incorporated multiple segments (DIMS) spectacle lenses will be prescribed in atropine plus optical defocus (ATD) treatment group while single vision spectacle lenses will be given in atropine only (AT) group. Cycloplegic refraction and axial lengths will be monitored every 6 months over 18-month study period. The primary outcomes are changes in cycloplegic refraction and axial lengths relative to the baseline over the study period.

**Discussion:**

The result will examine the combination effect of low dose atropine and myopic defocus on myopia control in a randomized controlled study. The findings will also explore the potential benefits of applying 0.01% atropine twice per day on myopic control and its potential side effects.

## Introduction

Myopia is characterized by an excessive elongation of the eyeball, leading to blurred distance vision. Its prevalence has reached high levels, especially in Asian regions such as China, Hong Kong, Taiwan, and Singapore [1]. Nearly half of the global population could be affected by 2050, with around 940 million individuals likely to exhibit high myopia (myopia ≤ −5.00D) [2]. Although blurry vision can be corrected by spectacles, contact lenses, or refractive surgery, they do not mitigate the risk of associated complications, which are more prevalent in high myopes. These include glaucoma [3], retinal detachment, and macular degeneration [4], all of which significantly impair visual performance and quality of life and contribute to myopia’s recognition as a global public health issue. The economic impact related to myopia is substantial. The direct costs of myopia for adults over 40 years were estimated at around USD 755 million annually in Singapore [5].

Managing myopia effectively and at an early stage is crucial to curbing its prevalence and the ensuing health and economic burdens. Current clinical practices employ both optical and pharmaceutical interventions for myopia control. Optical methods, such as inducing myopic defocus using positive lenses, have shown promise in slowing eye growth [6]. This idea has been supported by animal studies [7, 8] and clinical trials with myopic children using specially designed contact lenses and spectacle lenses [9, 10]. These interventions reduced myopia progression by 50–60% compared to the control group.

Pharmaceutical approaches, such as atropine eyedrops, have also proven effective. High-dose atropine (1%) significantly halted progression but accompanying with side effects like blurry near vision and photophobia [11]. Conversely, low dose atropine ranging from 0.01% to 0.05% has demonstrated a dose-dependent effect on slowing myopia, with minimal side effects [12].

Despite these interventions, myopia progression remains uncurbed in some cases, particularly in younger children [13]. Hence, there is a pressing need for more effective treatments that could potentially stop myopia progression entirely and prevent the onset of high myopia.

In this study protocol, we seek to combine two existing interventions—myopic defocus through optical means and low-dose atropine—to enhance control over myopia progression. Myopic defocus is delivered by the defocus incorporated multiple segments (DIMS) spectacle lenses. A +3.5D myopic defocus will be applied through DIMS lenses. We propose a novel combination of DIMS spectacle lens with low-dose atropine (0.01%) that is administered twice per day. This is a regimen not yet studied for effectiveness in controlling myopia progression. The aim is to observe the potential benefits of applying 0.01% atropine twice per day and optical defocus in myopia control among schoolchildren, while minimizing the side effects commonly associated with higher concentrations of atropine, such as blurry near vision and photophobia.

## Methods

### Study design and ethical approval

This is a parallel group, single-blinded, randomized, active-control trial. The study has been reviewed and approved by Institutional Review Board of The Hong Kong Polytechnic University and Institutional Review Board of the University of Hong Kong (HSEARS20221122002-01). The study has been registered on ClinicalTrials.gov (NCT identifier: NCT06358755). All the procedures will be carried out according to the guideline for Good Clinical Practice. Study personnel including administrative staff and investigators have attained a certificate of completion of the course on Good Clinical Practice.

### Participants: Inclusion and exclusion criteria

Schoolchildren will be recruited with the following inclusion criteria.

- Age at enrolment: 7–12 years
- Ethnicity: Hong Kong Chinese
- Myopia: -0.75DS (in spherical equivalent) or more in both eyes
- Astigmatism: -1.50DC or less in both eyes
- Difference in refraction between two eyes: 1.50D or less (in spherical equivalent)
- Best corrected monocular visual acuity (VA): 0.04 logMAR or better
- Ocular health: No abnormalities in both internal and external ocular health
- Systemic health: No abnormalities such as cardiac and respiratory diseases
- Binocular vision: No strabismus, diplopia, suppression and other binocular abnormalities
- Normal colour vision
- No previous refractive surgery or use of myopic control interventions, such as atropine, orthokeratology, and specialized spectacle lenses and contact lenses for myopic control
- Able to wear the prescribed spectacles
- No known allergy to atropine

### Exclusion criteria

Schoolchildren who meets any of the following criteria will be excluded from participation in this study, including prior myopia control treatment including orthokeratology, defocus soft contact lenses, progressive addition lenses, bifocal lenses, myopia control lenses, atropine and red light therapy, previous or current participation in myopia control studies, presence of eye disease or binocular vision problems including strabismus, amblyopia, oculomotor nerve palsies, corneal diseases, myopic macular degeneration and posterior staphyloma, colour vision deficiency, prior ocular or corneal surgery, having any ocular and systemic diseases and abnormalities that may affect visual function, refractive development, having systemic disease that are contradictory to atropine, or known allergy to cyclopentolate hydrochloride or atropine.

### Sample size calculation

In the study regarding low dose atropine for myopia control, mean change in refractive error after one year was 0.64 ± 0.56D (mean ± SD) [14]. Our previous randomized controlled trial showed DIMS slowed down myopia progression by approximately 60% [10]. The sample size is calculated based on an assumption that, adopting atropine and DIMS together will result in a 60% reduction of mean refractive error change relative to using only atropine, the difference detected between two groups (atropine alone vs. atropine and DIMS) will be 0.384 (0.64 x 60% = 0.384), and the effect size will be 0.685 (0.384/0.56 = 0.685). According to power analysis (G*Power Version 3.1.9.2), 46 subjects are required per group to achieve 90% power with a significance level of 0.05 (two-tailed). Assuming the dropout rate will be 20%, 56 subjects will be required for each group in this proposal. Therefore, 112 subjects (56 subjects × 2 groups) will be required for the entire study.

### Randomization and blinding

Eligible schoolchildren will be randomly allocated in one-to-one ratio using the block size of 4 to the atropine only (AT) or atropine plus optical defocus (ATD) treatment groups after the baseline measurement. AT group will receive 0.01% atropine (twice per day: 1 drop in the morning and 1 drop at night before bedtime) and single vision spectacle lens. ATD group will receive 0.01% atropine (twice per day: 1 drop in the morning and 1 drop at night before bedtime) and DIMS spectacle lens.

Masking participants and their parents is not feasible because of the unique pattern of DIMS spectacle lenses. Only the investigators who perform data collection of primary outcomes will be masked from grouping. Therefore, it is a single-blinded controlled trial.

### Interventions

Eligible schoolchildren will either receive low dose atropine (0.01%) plus single vision spectacle lenses or low dose atropine (0.01%) plus DIMS spectacle lenses. Low dose atropine eyedrops (0.01%) will be delivered in single-use unit dose as a sterile topical ophthalmic solution and free of preservatives. The volume of each unit dose is 0.5ml in polyethylene plastic bottle. Eyedrops will be provided to their parents/guardians for application of eyedrops twice per day. One drop is applied in the morning and one drop is applied at night before bedtime for 18 months. Spectacle lenses either single vision lenses or DIMS lenses will be prescribed to the schoolchildren according to the grouping. The prescription of the spectacle lenses for both groups will be updated if there is an increase in myopia of −0.50D or more or habitual visual acuity equal to or worse than 6/9 at 6^th^ and 12^th^-month follow up visit.

### Outcomes and measurements

The primary outcome measures are changes in cycloplegic refraction in spherical equivalent and axial length over 18 months from baseline. Cycloplegic refraction will be performed with the help of one to two drops of 1% cyclopentolate hydrochloride at baseline and every 6 months till 18 months. Accommodative amplitude will be examined after 30 minutes of cycloplegia. An extra drop of 1% cyclopentolate hydrochloride will be instilled if the amplitude of accommodation is more than +2.00D. Cycloplegic refraction will be measured by an open-field autorefractor (Shin-Nippon NVision-K5001, Japan). Axial length will be measured by an optical biometer (IOLMaster500, Carl Zeiss, Germany). Five measurements will be taken and averaged.

The secondary outcome measures include accommodative amplitude and responses, pupil size and other ocular biometrics such as choroidal thickness, which will be collected over the study period.

### Procedures

Participants will be recruited via university mass email, posters and social media. An onsite screening will be carried out to confirm their eligibility. Consent and assent will be obtained from both parents and schoolchildren before data collection of basement measurement. Baseline measurement includes cycloplegic refraction, axial length and other ocular biometry, accommodative amplitude and responses, pupil size, intraocular pressure, external and internal ocular health examination. Cycloplegic refraction will be measured by an open-field autorefractor (Shin-Nippon NVision-K5001, Japan). Axial length will be measured by an optical biometer (IOLMaster500, Carl Zeiss, Germany). Other ocular biometry such as corneal curvature, corneal thickness, anterior chamber depth, lens thickness will be measured using Lenstar LS900 (Haag-Streit, Switzerland).

**Fig 1:**
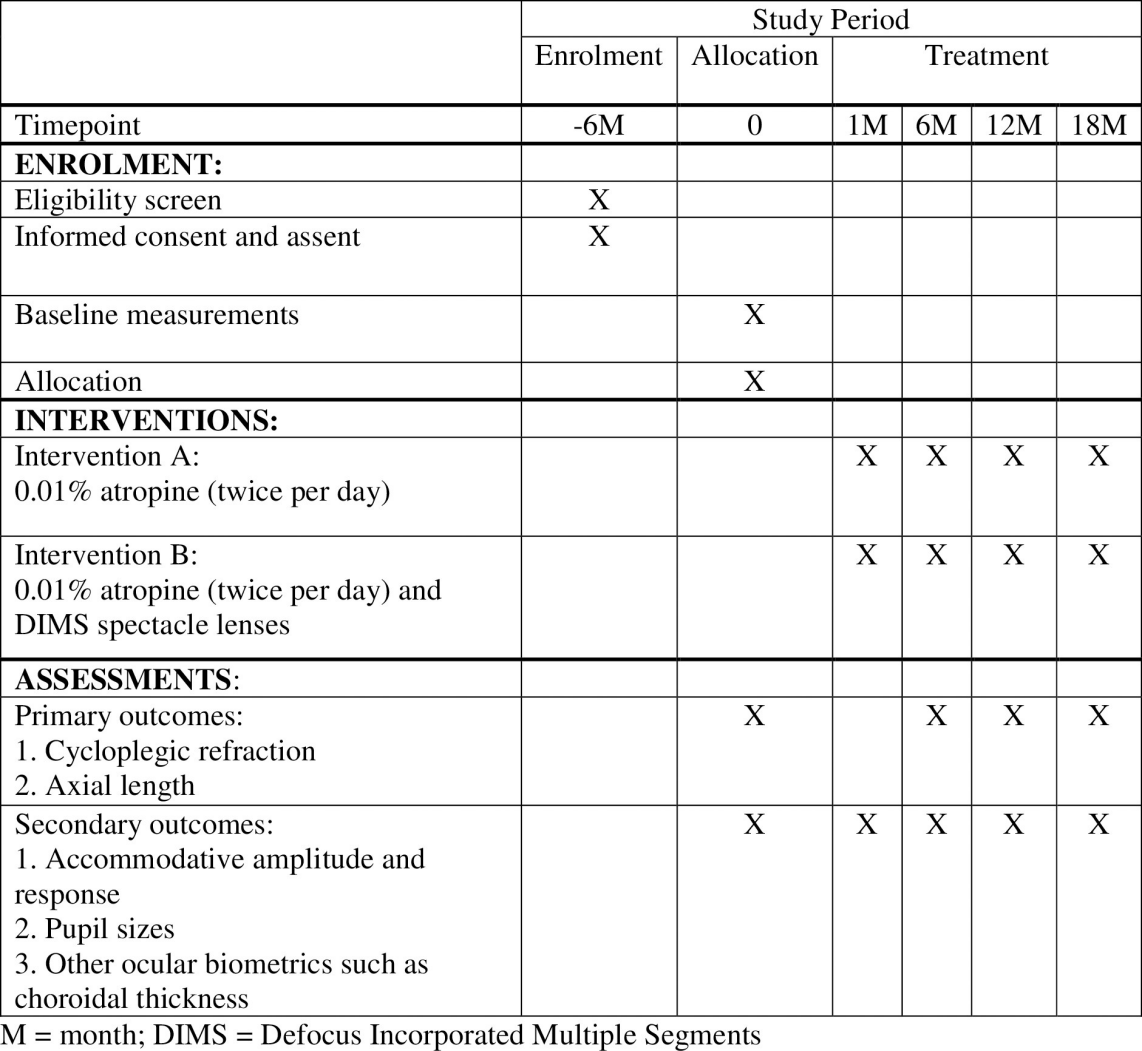
SPIRIT schedule of enrolment, interventions and assessments.

Enrolled schoolchildren will be randomly allocated into either the atropine only (AT) or atropine plus optical defocus (ATD) treatment groups after the baseline measurement in one-to-one ratio. Spectacle prescription will be placed according to their grouping. AT group will receive a pair of single vision spectacle lenses while ATD group will receive a pair of DIMS spectacle lenses. Both groups will receive 0.01% atropine from ophthalmologists. A follow up will be scheduled after one-month usage of atropine to evaluate the presence of any adverse effects related to atropine (Fig 1). Cycloplegic refraction and other measurements will be performed at 6^th^, 12^th^ and 18^th^-month during the study period. Data management plan such as the detailed procedures of data collection and case report forms, data entry procedures, data management and report and handling of sensitive data in this clinical trial has been established in clinic protocol (S1 Checklist).

### Historical control

A placebo control group is not feasible and ethical when considering the existence of established treatments for myopia control. One of the objectives in this study is to evaluate if combination therapy is superior to single-modality treatment. To compare the efficacy of the proposed intervention, a historical control will be derived from the patient records at The Hong Kong Polytechnic University Optometry Clinic. The selection will include data from individuals who conform to the eligibility criteria of the current study and have a follow-up period of no less than 18 months. The patients included in historical control only received single vision spectacle lenses over the period.

### Compliance

To ensure the compliance of the usage of atropine, an access to an online platform will be given to the parents/guardians to record the usage of eyedrops. Compliance of spectacle lens wear will be recorded by self-reporting in the follow-up visit.

### Safety

A set of guidelines are established to report the adverse event over the study period. Any event involves abnormal clinical findings, symptoms or diseases associated to the use of interventions in the participants, whether or not considered as intervention-related will be reported and recorded. Information to be collected includes event description, time of onset, clinician’s assessment of severity, relationship to interventions and time of resolution or stabilization of the event. All adverse events will be followed to adequate resolution and they will be judged by the Study Management Committees to decide if the participants are required to withdraw from the study.

Blurry vision at near and photophobia are commonly experienced in children with low dose atropine. Progressive addition lenses or photochromatic lenses will be prescribed to the participants if these side effects persist.

### Statistical analysis

Five readings of cycloplegic refraction and axial lengths will be averaged in right eyes at each time point. Cycloplegic refraction will be presented by spherical equivalent that is the sum of spherical power and half of the cylindrical power. At baseline measurement, all the parameters including basic demographics, refractive errors, and ocular biometrics will be compared between two groups using unpaired t-test. The normality of the data will be examined using Kolmogorov-Simrnov test.

Changes in refraction, axial length, pupil sizes, accommodative responses, and other ocular biometrics at different time points will be calculated relative to the baseline. Changes will then be compared between two groups using mixed repeated measures analysis of covariance controlled by age, baseline myopia and parental myopia. Factors such as baseline refraction and gender that could be associated with myopia control efficacy will be analysed using multivariate logistic regression.

All statistical analyses will be conducted using IBM SPSS statistical software (version 29.0). A level of significance of 0.05 will be used.

Myopia progression over 18 months in two groups will be calculated as the difference between refraction at the baseline and the 18-month visit. The efficacy of myopia control of each group will be calculated by dividing the difference in myopia progression between the atropine group/the atropine plus DIMS group, and the historical control group with the myopia progression in the atropine group, then multiplied by 100%.

(Efficacy of atropine or atropine plus DIMS = Difference in myopia progression after the treatment/ myopia progression in historical control group) × 100%)

## Discussion

This study will be the first randomized controlled study to investigate the combination effect of optical defocus and low dose atropine on myopia control in schoolchildren. Optical and pharmaceutical interventions have been adopted in clinical practices to control myopia progression in schoolchildren [6]. However, the effectiveness varies between individuals and it did not completely halt the myopia progression. To achieve higher effectiveness in controlling myopia progression, combination therapy that using both optical and pharmaceutical paradigms has been investigated because the underlying mechanism involved in optical and pharmaceutical interventions might be different. Schoolchildren who received orthokeratology and low dose atropine demonstrated a smaller axial elongation when combined to those with orthokeratology or low dose atropine alone [15, 16]. Therefore, our study is to investigate the combination effect of DIMS spectacle lenses (that casts a myopic defocus) and low dose atropine on myopia progression. Although there were two studies developed combination therapy of DIMS spectacle lenses and atropine [17, 18], their study designs may not be fair enough to conclude the combination therapy is more beneficial than the single interventions. Both studies were not randomized controlled trials and only 1-year study was carried out. Therefore, there is a need to conduct a randomized controlled trial to study the combination therapy of low dose atropine and DIMS lenses. The paradigm of 0.01% atropine is also novel compared to the previous study. Low dose atropine (0.01%) will be administered twice per day. This is to explore if this paradigm in eyedrop administration could improve the effectiveness of myopia control with a minimal adverse effect such as blurry near vision and photophobia.

There are some limitations of our study protocol. The study duration is 18 months that is shorter than the usual study due to the budget constraints. We anticipated that the myopic control effect could be observed after 6 to 12 months of treatment based on our previous DIMS study. Therefore, the aim of the current study could still be addressed using an 18-month clinical trial. Apart from the study duration, one of the weakness is the absence of control group due to the ethical considerations. Since both DIMS lenses and low dose atropine has been proven to significantly control myopia progression, it is not feasible to recruit participants who received placebo as control. To improve this weakness, a historical control group with the same inclusion criteria will be extracted from the University-based optometry clinic. The changes in refractive errors and axial length in this control group can be extracted for calculating the efficacy of atropine or atropine plus DIMS lenses on myopia progression.

## Conclusion

This study will contribute to our understanding of the effect of combining optical defocus and low dose atropine on myopia control in schoolchildren. The outcome would directly benefit myopic patients by reducing their risk to develop high myopia. The results will also provide eyecare professionals an evidence-based options to better manage the myopic population.

## Supporting information

S1 Checklist SPIRIT 2013 checklist: Recommended items to address in a clinical trial protocol and related documents*. (PDF)

S1 File (DOCX)

## References

[1] Morgan IG, Ohno-Matsui K, Saw SM. Myopia. Lancet. 2012;379(9827):1739–48. doi: 10.1016/S0140-6736(12)60272-4 . 22559900

[2] Holden BA, Fricke TR, Wilson DA, Jong M, Naidoo KS, Sankaridurg P, et al. Global Prevalence of Myopia and High Myopia and Temporal Trends from 2000 through 2050. Ophthalmology. 2016;123(5):1036–42. doi: 10.1016/j.ophtha.2016.01.006 . 26875007

[3] Mitchell P, Hourihan F, Sandbach J, Wang JJ. The relationship between glaucoma and myopia: the Blue Mountains Eye Study. Ophthalmology. 1999;106(10):2010–5. doi: 10.1016/s0161-6420(99)90416-5 . 10519600

[4] Saw SM, Gazzard G, Shih-Yen EC, Chua WH. Myopia and associated pathological complications. Ophthalmic Physiol Opt. 2005;25(5):381–91. doi: 10.1111/j.1475-1313.2005.00298.x . 16101943

[5] Zheng YF, Pan CW, Chay J, Wong TY, Finkelstein E, Saw SM. The economic cost of myopia in adults aged over 40 years in Singapore. Invest Ophthalmol Vis Sci. 2013;54(12):7532–7. doi: 10.1167/iovs.13-12795 . 24159089

[6] Jonas JB, Ang M, Cho P, Guggenheim JA, He MG, Jong M, et al. IMI Prevention of Myopia and Its Progression. Invest Ophthalmol Vis Sci. 2021;62(5):6. doi: 10.1167/iovs.62.5.6 . 33909032 PMC8083117

[7] Tse DY, Lam CS, Guggenheim JA, Lam C, Li KK, Liu Q, et al. Simultaneous defocus integration during refractive development. Invest Ophthalmol Vis Sci. 2007;48(12):5352–9. doi: 10.1167/iovs.07-0383 . 18055781

[8] McFadden SA, Tse DY, Bowrey HE, Leotta AJ, Lam CS, Wildsoet CF, et al. Integration of defocus by dual power Fresnel lenses inhibits myopia in the mammalian eye. Invest Ophthalmol Vis Sci. 2014;55(2):908–17. doi: 10.1167/iovs.13-11724 . 24398103 PMC3926275

[9] Lam CS, Tang WC, Tse DY, Tang YY, To CH. Defocus Incorporated Soft Contact (DISC) lens slows myopia progression in Hong Kong Chinese schoolchildren: a 2-year randomised clinical trial. Br J Ophthalmol. 2014;98(1):40–5. doi: 10.1136/bjophthalmol-2013-303914 . 24169657 PMC3888618

[10] Lam CSY, Tang WC, Tse DY, Lee RPK, Chun RKM, Hasegawa K, et al. Defocus Incorporated Multiple Segments (DIMS) spectacle lenses slow myopia progression: a 2-year randomised clinical trial. Br J Ophthalmol. 2020;104(3):363–8. doi: 10.1136/bjophthalmol-2018-313739 . 31142465 PMC7041503

[11] Chua WH, Balakrishnan V, Chan YH, Tong L, Ling Y, Quah BL, et al. Atropine for the treatment of childhood myopia. Ophthalmology. 2006;113(12):2285–91. doi: 10.1016/j.ophtha.2006.05.062 . 16996612

[12] Yam JC, Jiang Y, Tang SM, Law AKP, Chan JJ, Wong E, et al. Low-Concentration Atropine for Myopia Progression (LAMP) Study: A Randomized, Double-Blinded, Placebo-Controlled Trial of 0.05%, 0.025%, and 0.01% Atropine Eye Drops in Myopia Control. Ophthalmology. 2019;126(1):113–24. doi: 10.1016/j.ophtha.2018.05.029 . 30514630

[13] Moore M, Lingham G, Flitcroft DI, Loughman J. Myopia progression patterns among paediatric patients in a clinical setting. Ophthalmic Physiol Opt. 2024;44(2):258–69. doi: 10.1111/opo.13259 . 38062894

[14] Chia A, Chua WH, Cheung YB, Wong WL, Lingham A, Fong A, et al. Atropine for the treatment of childhood myopia: safety and efficacy of 0.5%, 0.1%, and 0.01% doses (Atropine for the Treatment of Myopia 2). Ophthalmology. 2012;119(2):347–54. doi: 10.1016/j.ophtha.2011.07.031 . 21963266

[15] Tan Q, Ng AL, Choy BN, Cheng GP, Woo VC, Cho P. One-year results of 0.01% atropine with orthokeratology (AOK) study: a randomised clinical trial. Ophthalmic Physiol Opt. 2020;40(5):557–66. doi: 10.1111/opo.12722 . 32776533

[16] Tang T, Lu Y, Li X, Zhao H, Wang K, Li Y, et al. Comparison of the long-term effects of atropine in combination with Orthokeratology and defocus incorporated multiple segment lenses for myopia control in Chinese children and adolescents. Eye (Lond). 2024. doi: 10.1038/s41433-024-02987-5 . 38418604 PMC11156845

[17] Huang Z, Chen XF, He T, Tang Y, Du CX. Synergistic effects of defocus-incorporated multiple segments and atropine in slowing the progression of myopia. Sci Rep. 2022;12(1):22311. doi: 10.1038/s41598-022-25599-z . 36566245 PMC9789944

[18] Nucci P, Lembo A, Schiavetti I, Shah R, Edgar DF, Evans BJW. A comparison of myopia control in European children and adolescents with defocus incorporated multiple segments (DIMS) spectacles, atropine, and combined DIMS/atropine. PLoS One. 2023;18(2):e0281816. doi: 10.1371/journal.pone.0281816 . 36795775 PMC9934319

