# Supplementary material for: Combination effect of optical defocus and low dose atropine in myopia control: Study protocol for a randomized clinical trial
Source: PLoS One. 2024 Jun 26;19(6):e0306050. doi: 10.1371/journal.pone.0306050 (PMC11206955; doi:10.1371/journal.pone.0306050)
Supplement: S1 File — (DOCX) [file pone.0306050.s002.docx]

**Combination Effect of Optical Defocus and Low Dose Atropine in Myopia Control – a Randomized Clinical Trial**

**PI: Dr Ka Man Chun**

**Co-I: Prof Chi Ho TO, Prof Christopher Kai Shun Leung, Dr Yan Yin TSE and Dr Sheung Shun NG**

**School of Optometry, The Hong Kong Polytechnic University**

**and**

**Department of Ophthalmology, The University of Hong Kong**

**Version Number: v 1.1**

**18 Jan 2024**

| Affected Section (s) | Summary of Revisions Made | Rationale |
| --- | --- | --- |
| p.6 | Update the post of Prof Chi Ho To |  |
| Section 1 p.3, section 2 p.9, section 5.14 p.19 | The frequency of 0.01% atropine is changed to twice per day | To align with the approved study protocol from Bureau |
| Section 5 p.15 | Inclusion of Provain-POS 0.5% as the topical anaesthetic | Provain-POS 0.5% shares the same indication and precautious after use. Provain-POS 0.5% can be stored under room temperature for better management |

**Summary of changes from previous version 1.0:**

**Content**

[1. Overview 1](#_Toc164344867)

[2. All items from the World Health Organization Trial Registration Data Set 6](#_Toc164344868)

[3. Study Plan Schematic 8](#_Toc164344869)

[4. Background and Rationale for Study 9](#_Toc164344870)

[4.1 Population of Myopia and Its Influence 9](#_Toc164344871)

[4.2 Current Myopia Control Interventions and Problems 9](#_Toc164344872)

[4.2.1 Atropine 9](#_Toc164344873)

[4.2.2 DIMS lenses 10](#_Toc164344874)

[4.3 Combination of myopia control interventions 10](#_Toc164344875)

[5. Study Objective 12](#_Toc164344876)

[6.1 Inclusion Criteria 12](#_Toc164344877)

[6.2 Exclusion Criteria 12](#_Toc164344878)

[6.3 Recruitment 12](#_Toc164344879)

[6.4 Study Intervention 13](#_Toc164344880)

[6.4.1 Atropine 13](#_Toc164344881)

[6.4.2 Spectacles 14](#_Toc164344882)

[6.5 Randomization 14](#_Toc164344883)

[6.6 Blinding 14](#_Toc164344884)

[6.7 Withdrawal Criteria 15](#_Toc164344885)

[6.8 Lost to Follow-up 16](#_Toc164344886)

[6.9 Eligibility and Baseline Assessment 16](#_Toc164344887)

[6.10 Primary Outcome Measure 18](#_Toc164344888)

[6.11 Secondary Outcome Measure 18](#_Toc164344889)

[6.12 Schedule of Intervention and Follow-up 19](#_Toc164344890)

[6.13 Data Collection Schedules 20](#_Toc164344891)

[6.13.1 Eligibility, baseline assessments and randomization 20](#_Toc164344892)

[6.13.2 Spectacles and atropine dispensing 21](#_Toc164344893)

[6.13.3 Follow-up assessments (data related to treatment outcomes) 21](#_Toc164344894)

[6.14 Protocol Violations 21](#_Toc164344895)

[7. Statistical Considerations 22](#_Toc164344896)

[7.1 Sample Size 22](#_Toc164344897)

[7.2 Statistical analysis 22](#_Toc164344898)

[7.2.1 Baseline characteristics 22](#_Toc164344899)

[7.2.2 Treatment effects 22](#_Toc164344900)

[7.2.3 Tolerability 23](#_Toc164344901)

[7.2.4 Procedures to account for missing data 23](#_Toc164344902)

[7.2.5 Interim analyses 23](#_Toc164344903)

[7.3 Data management 23](#_Toc164344904)

[7.3.1 Source data 23](#_Toc164344905)

[7.3.2 Data Recording and record keeping 24](#_Toc164344906)

[8. Ethical Approval and Consent 24](#_Toc164344907)

[8.1 Ethics Approval 24](#_Toc164344908)

[8.2 Informed Consent 25](#_Toc164344909)

[9. Assessment of Safety/Adverse Event Reporting 26](#_Toc164344910)

[9.1 Adverse Events 26](#_Toc164344911)

[9.1.1 Definition of an Adverse Event 26](#_Toc164344912)

[9.1.2 Contact for Notification of Adverse Events 26](#_Toc164344913)

[9.2 Serious Adverse Events 27](#_Toc164344914)

[9.2.1 Definition of a Serious Adverse Event 27](#_Toc164344915)

[9.2.2 Significant Adverse Events 28](#_Toc164344916)

[9.2.3 Non-Significant Adverse Events 28](#_Toc164344917)

[9.2.4 Contact for notification of serious adverse events 29](#_Toc164344918)

[9.3 Reporting safety information 29](#_Toc164344919)

[9.4 Unblinding 29](#_Toc164344920)

[9.5 Data safety and monitoring 30](#_Toc164344921)

[10. Intervention supplies 31](#_Toc164344922)

[10.1 Study treatment identification 31](#_Toc164344923)

[10.2 Handing and Dispensing of Study Treatment 31](#_Toc164344924)

[10.3 Packaging and Labelling 31](#_Toc164344925)

[10.3.1 Spectacle Lenses 31](#_Toc164344926)

[10.3.2 Atropine 31](#_Toc164344927)

[10.4 Treatment Supply Records 31](#_Toc164344928)

[11. Relevance to Health 33](#_Toc164344929)

[12. Dissemination of Results 35](#_Toc164344930)

[12.1 Trial Registration 35](#_Toc164344931)

[12.2 Study Participants 35](#_Toc164344932)

[12.3 Academic / Professional Colleagues 35](#_Toc164344933)

[13. Administrative Section 36](#_Toc164344934)

[13.1 Adherence to Protocol 36](#_Toc164344935)

[13.2 Protocol Revision Procedures 36](#_Toc164344936)

[13.3 Case report form procedures 36](#_Toc164344937)

[13.4 Monitoring / Source Document Verification 37](#_Toc164344938)

[13.5 Data Confidentiality and Security 37](#_Toc164344939)

[13.6 Reporting Schedule 37](#_Toc164344940)

[13.7 Record Retention Policy 37](#_Toc164344941)

[13.8 Insurance 38](#_Toc164344942)

[13.9 Ownership of Data and Publication Policy 38](#_Toc164344943)

[13.10 Declaration of interests 38](#_Toc164344944)

[14. Abbreviations 39](#_Toc164344945)

[15. References 41](#_Toc164344946)

[16 Appendices 43](#_Toc164344947)

**Study Title**

Combination Effect of Optical Defocus and Low Dose Atropine in Myopia Control – a Randomized Clinical Trial

**Short Title**

Myopia control using DIMS and low dose atropine

**Principal Investigator**

Dr Ka Man CHUN (Research Assistant Professor, School of Optometry)

**Co-Investigators**

Prof Chi Ho TO (Visiting Chair Professor, School of Optometry)

Prof Christopher Kai Shun LEUNG (Department Chairperson and Clinical Professor, Department of Ophthalmology, The University of Hong Kong)

Dr Yan Yin TSE (Associate Professor, School of Optometry)

Dr Sheung Shun NG (Associate Consultant Optometrist, School of Optometry)

**Steering Committee Members:**

Dr Ka Man CHUN (Chair), Prof Christopher Kai Shun LEUNG (Co-I) and Dr Dennis Yan-Yin Tse (Co-I)

**Study Management Committee Members:**

Dr Ka Man CHUN (PI), Prof Christopher Kai Shun LEUNG (Co-I) and Prof Chi Ho TO (Co-I)

**Study Centre**

1. Centre of Myopia Research, School of Optometry, The Hong Kong Polytechnic University
2. HKU Eye Centre, Department of Ophthalmology, The University of Hong Kong

# 1. Overview

**Title of Study**

Combination Effect of Optical Defocus and Low Dose Atropine in Myopia Control – a Randomized Clinical Trial

**Short Title**

Myopia control using DIMS and low dose atropine

**Study Description and Methodology**

Prevalence of myopia is high locally and in some East Asian Regions such as China, Taiwan and Singapore (1). In Hong Kong, 60% of 12-year-old schoolchildren were myopic in a 2012 study (2), and prevalence of myopia is predicted to further increase with approximately 50% of the world population by 2050 and nearly 940 million people will have high myopia, which is defined as myopia ≤-5.00D (3). High myopia is associated with risk of sight-threatening ocular conditions including glaucoma, retinal detachment and macular degeneration (4, 5). These ocular problems cause visual impairments and lower living qualities of the patients (6). Moreover, high myopia related ocular complications induce financial burden on public health aspect.

There are several myopia control products available in clinical and commercial practices, which can be classified as optical and pharmaceutical interventions (7). Optical interventions make use of myopic defocus to slow down myopia progression. Myopic defocus can be generated by Defocus Incorporated Multiple Segments (DIMS) with positive-powered lens embedded to induce focal planes in front of retina and to slow eye growth. It is found that DIMS lenses slowed down myopia progression by 60% comparing with no intervention was adopted (8). Pharmaceutical intervention, known as atropine, was proven to be effective to control myopia. Low dose atropine with concentration ranging from 0.01% and 0.05% slowed down myopia progression by 20 and 60% respectively (9-11).

However, none of the currently available interventions completely stop myopia progression and their effectiveness varies between individuals, which younger schoolchildren was found to be less powerful (12). Therefore, it is essential to explore treatment strategies that have potential in increasing effectiveness and may ideally halt myopia progression. Based on these considerations, the aim of this study is to investigate if combination of atropine and DIMS lenses increases the ability to control myopia in schoolchildren than atropine is used alone.

**Objective**

To investigate the effect of combination of optical defocus and low dose atropine in myopia control

**Study Population**

A total of 112 Hong Kong Chinese schoolchildren aged between 7 to 12 will be recruited in Optometry Research Clinic of The Hong Kong Polytechnic University. The schoolchildren are categorized into two groups: one group with single vision spectacles and 0.01% atropine (AT Group); another group with optical defocus spectacles and 0.01% atropine (ATD Group), with 56 subjects in each group. They must not have had prior or current myopia control treatment, have no ocular or systemic diseases or abnormalities that affect visual function, refractive development or spectacle lens wear. No previous intraocular or corneal surgery has been done. They must not be allergic to atropine.

**Site of the study**

- Centre of Myopia Research, School of Optometry, The Hong Kong Polytechnic University
- HKU Eye Centre, Department of Ophthalmology, The University of Hong Kong (Address:

**Inclusion Criteria**

- Age at enrollment: 7 to 12 years
- Ethnicity: Hong Kong Chinese
- Myopia: -0.75D (in spherical equivalent) or above in both eyes
- Astigmatism: -1.50D or less in both eyes
- Anisometropia: 1.50D or less (in spherical equivalent) between two eyes
- Best corrected monocular visual acuity (VA): 0.04 logMAR or better
- Ocular health: No abnormalities in both internal and external ocular health
- Systemic health: No abnormalities such as cardiac and respiratory diseases
- Binocular vision: No strabismus, diplopia, suppression and other binocular abnormalities
- Normal colour vision
- No previous refractive surgery or use of myopic control interventions, such as atropine, orthokeratology, and specialized spectacle lenses and contact lenses for myopic control
- Able to wear the prescribed spectacles
- No known allergy to atropine

**Exclusion Criteria**

- Eye disease or binocular vision problems (e.g., strabismus, amblyopia, oculomotor nerve palsies, corneal disease, etc.)
- Previous intraocular or corneal surgery
- Colour vision deficiencies
- Systemic disease that may affect vision, vision development (e.g. diabetes mellitus, hypertension, Down syndrome, etc.)
- Systemic disease that are contradictory to atropine (e.g. asthma, cardiac diseases, etc.)
- Previous gas permeable, soft bifocal, or orthokeratology contact lenses wear or bifocal / progressive addition lens spectacles wear or use of atropine or pirenzepine (longer than one month of usage)
- Previous or current participation in myopia control studies
- Allergy to cyclopentolate hydrochloride or atropine

**Randomization**

Eligible participants will be randomly allocated in one-to-one ratio to the atropine only (AT) or atropine plus optical defocus (ATD) treatment groups.

**Experimental groups**

AT group: 0.01% atropine (twice per day: 1 drop in the morning and 1 drop at night before bedtime) and single vision spectacle lens

ATD group: 0.01% atropine (twice per day: 1 drop in the morning and 1 drop at night before bedtime) and DIMS spectacle lens

**Criteria for Evaluation**

*Primary outcomes*

The primary outcome measures are changes in cycloplegic refraction in spherical equivalent and axial length over 18 months from baseline.

*Secondary outcomes*

The secondary outcome measures are accommodative amplitude and response, pupil size, other ocular biometrics, treatment compliance, and adverse events and intervention events at all visits.

Other optometric outcomes not listed here will be considered exploratory in nature.

**Statistical Methods**

*Sample Size Calculation*

In the study regarding low dose atropine for myopia control, mean change in refractive error after one year was 0.64 ± 0.56D (mean ± SD) (10). Our previous randomized clinical trial showed DIMS slowed down myopia progression by approximately 60% (8). The sample size is calculated based on an assumption that, adopting atropine and DIMS together will result in a 60% reduction of mean refractive error change relative to using only atropine, the difference detected between two groups (atropine alone vs. atropine and DIMS) will be 0.384 (0.64 x 60% = 0.384), and the effect size will be 0.685 (0.384/0.56 = 0.685). According to power analysis (G*Power Version 3.1.9.2), 46 subjects are required per group to achieve 90% power with a significance level of 0.05 (two-tailed). Assuming the dropout rate will be 20%, 56 subjects will be required for each group in this proposal. Therefore, 112 subjects (56 subjects × 2 groups) will be required for the entire study.

As the quantitative effectiveness of DIMS combined with atropine is not known, two conservative assumptions are made during the sample size calculation: standard deviations are the same in the atropine plus DIMS group relative to the atropine alone group; and the previous study considering atropine tracked a 12-month change, while the proposed study treatment period will be 18 months.

*Statistical Analysis*

Data of right eyes will be used for analyses. Demographic and optometric data will be displayed as mean and standard deviation, or number and percentage as appropriate. The Chi-square test (or if appropriate Fisher’s Exact test) and unpaired t-test will be performed to compare baseline between-group differences in demographics (age, sex) and primary and secondary outcomes between the control group. Myopia progression over 18 months will be calculated as the difference between the primary outcomes (SER and AL) at the sixth and the first visits.

Unpaired t-test will be used to compare the changes in primary outcomes between two groups. If statistically significant differences in baseline demographic characteristics between groups are found, these significant covariates will also be adjusted in these primary analyses with Analysis of Covariates. In this case, both the unadjusted and adjusted results will be reported. Multiple regression analysis with the primary outcomes at the sixth visit will act as the dependent variable and independent variables, alongside with the secondary outcomes, will be employed to identify factors that may associated with myopia control effectiveness.

All analyses will use intention-to-treat analyses. Missing values of outcome variables and covariates will be replaced using multiple imputation procedure with 10 sets of imputations assuming missing at random. Sensitivity analyses for the primary outcomes include complete case analysis and per-protocol analysis. All analyses will be 2-tailed with a significance level of 5%.

The CONSORT Statement will guide the reporting of results.

**Funding**

Health and Medical Research Fund is the principle funder of this trial.

# 2. All items from the World Health Organization Trial Registration Data Set

Trial identifying number (Clinicaltrials.gov): NCT06358755

Date of registration: 20 March 2024

Source of monetary support: Health and Medical Research Fund

Sponsor: Health and Medical Research Fund from Health Bureau of the Hong Kong Special Administrative Region

Contact for public queries: Dr Rachel Ka Man Chun, PhD (+852-27664224, [rachel.chun@polyu.edu.hk](mailto:rachel.chun@polyu.edu.hk))

Contact for scientific queries: Dr Rachel Ka Man Chun, PhD, School of Optometry, The Hong Kong Polytechnic University

Public title: Combination Effect of Optical Defocus and Low Dose Atropine in Myopia Control

Scientific title: Combination Effect of Optical Defocus and Low Dose Atropine in Myopia Control – a Randomized Clinical Trial

Country of recruitment: Hong Kong SAR, China

Health condition or problem studied: Myopia

Interventions:

- 0.01% atropine (twice per day) plus Defocus Incorporated Multiple Segments spectacle lenses

- 0.01% atropine (twice per day)

Key inclusion and exclusion criteria

Inclusion criteria

- Age eligible for study: 7 to 12 years old
- Sexes eligible for study: both
- Myopia: -0.75D (in spherical equivalent) or above in both eyes
- Astigmatism: -1.50D or less in both eyes
- Anisometropia: 1.50D or less (in spherical equivalent) between two eyes
- Best corrected monocular visual acuity (VA): 0.04 logMAR or better
- Ocular health: No abnormalities in both internal and external ocular health
- Systemic health: No abnormalities such as cardiac and respiratory diseases
- Binocular vision: No strabismus, diplopia, suppression and other binocular abnormalities
- Normal colour vision

Exclusion criteria: Previous use of myopic control interventions, allergy to cyclopentolate hydrochloride or atropine, ocular and systemic diseases that affect vision and vision development, color vision deficiency, previous ocular surgery

Study type: Interventional/ Allocation: randomized/ Intervention model: parallel assignment/ Masking: single blind from investigators and outcome assessor/ Primary purpose: treatment, phase 2

Date of first enrolment: not yet start

Target sample size: 112

Recruitment status: not yet recruiting

Primary outcomes: Changes in cycloplegic refraction and changes in axial length

# 3. Study Plan Schematic


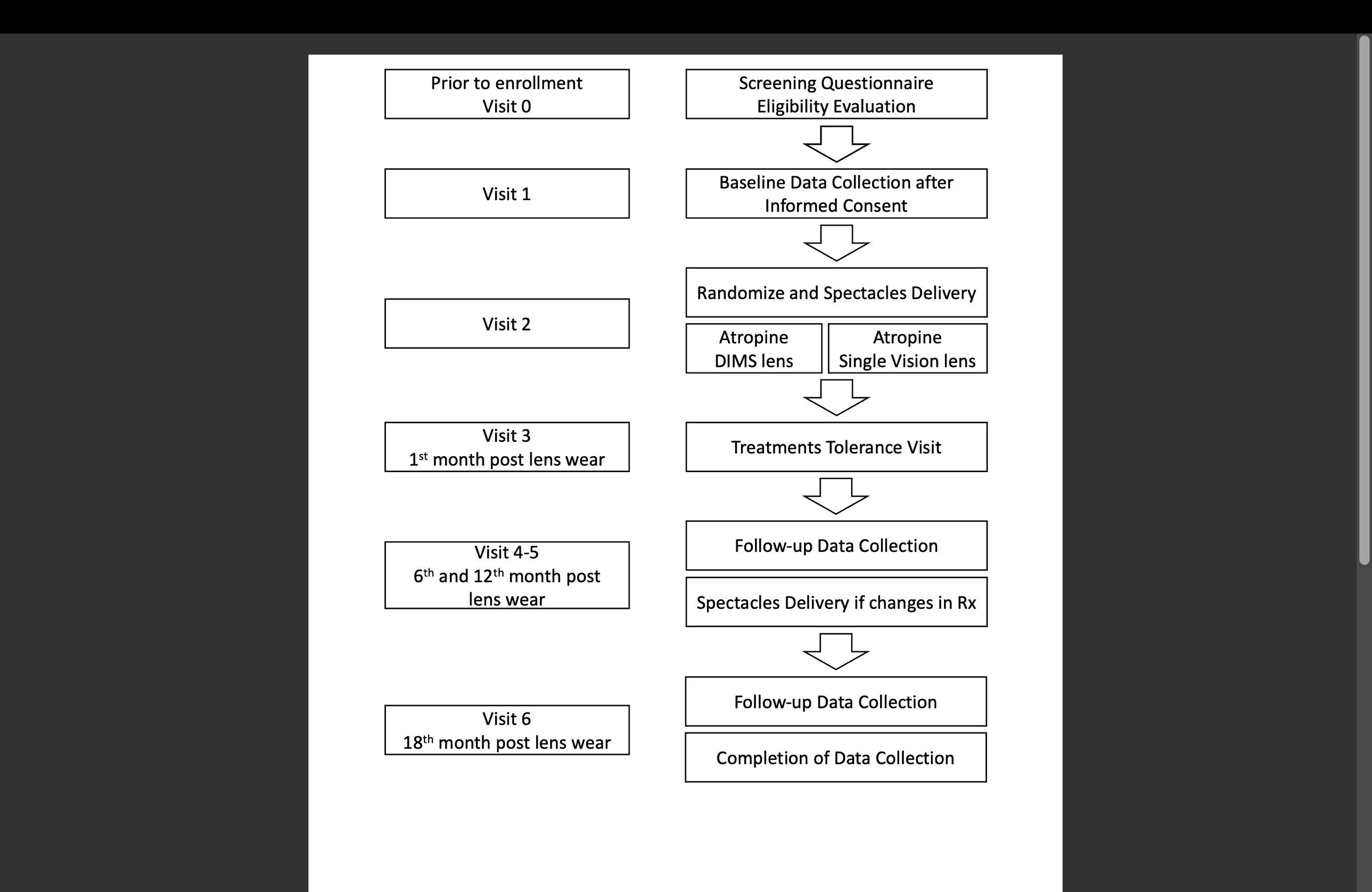


# 4. Background and Rationale for Study

## 4.1 Population of Myopia and Its Influence

Myopia, also known as shortsightedness, refers to an ocular condition characterized by excessive elongation of eyeball. It is common in South Asian regions including Hong Kong which affects more than 60% of local schoolchildren at the age of 12 (1, 2). The prevalence is predicted to rise, with approximately 50% of the world population projected to be myopic in 2050, including nearly 10% projected to have high myopia, which means the prevalence of myopia and high myopia in young adults is going to elevate in the coming decades as the children grow up (3). Although blurry vision at distance in myopes can be corrected by spectacles, contact lenses, or refractive surgery, these corrections do not reduce the risk of myopia-related ocular diseases and complications. Risks of sight-threatening ocular diseases as glaucoma and myopic macular degeneration increase with the magnitude of myopia (4, 5). These ocular problems significantly reduce quality of life of the patients (6). Myopia has also become a worldwide public health concern due to the financial burden associated with high myopia-related complications. In 2013, the total direct cost of myopia in adults over 40 years was estimated to be approximately USD$755 million per year in Singapore, where myopia prevalence is high (13).

## 4.2 Current Myopia Control Interventions and Problems

It is recommended to adopt myopia control interventions at early stage of life in an effective way in order to reduce high myopia prevalence and its related complication. A number of myopia control products are now available in clinical and commercial practice. They are broadly classified as optical and pharmaceutical interventions (7).

### 4.2.1 Atropine

Atropine is known as one of the effective pharmaceutical interventions used in controlling myopia progression. Topical administration of high dose atropine at a concentration of 1% was very potent in halting myopia progression, but it had significant adverse effects including blurry near vision, photophobia and hypersensitivity (9) which limited its usage. Low dose atropine, at concentrations ranging from 0.01% to 0.05%, also demonstrated a dose-dependent reduction of myopia (14), with 0.01% and 0.05% atropine reducing myopia progression by 20% and 60%, respectively (9-11). Adverse effects were also dose dependently associated with low dose atropine treatment. As atropine is an invasive option for controlling myopia, it might not be the preferred first line of treatment even though it is appropriate.

### 4.2.2 DIMS lenses

Defocus Incorporated Multiple Segments, DIMS lens is an optical method to control myopia progression (15). It adopts concept of myopic defocus, which a positive-powered lens relative to refractive error of the user is embedded, so the focal plane of the image is cast in front of the retina, which induces a “stop” signal for eye elongation. Myopic defocus has been shown to inhibit myopic eye growth in animal models including in chicks, guinea pigs, tree shrew, and monkeys (16). In DIMS lens, a +3.50D myopic defocus is incorporated in defocus incorporated multiple segments (DIMS) spectacle lenses. DIMS spectacle lenses consist of a central 9-mm optical zone with distant correction which provides a clear distance vision, and it is surrounded by multiple tiny spots of myopic defocus lenses (+3.50D) at periphery. Myopia progression was significantly retarded by about 60% in schoolchildren wearing DIMS spectacle lenses in a 2-year randomized controlled study comparing with no intervention is adopted (8). DIMS spectacle lenses are now commercially available as one of the non-invasive myopic control interventions.

However, none of the currently available intervention completely halts myopia progression and their effectiveness varies between individuals. It is found that the younger the schoolchildren they are, the less effective the myopic control interventions are (12). Therefore, it is crucial to explore treatment strategies that have potential for increased effectiveness and may completely halt myopia progression, thereby avoiding development of high myopia.

### 4.3 Combination of myopia control interventions

Monotherapy for myopia control is partially effective to slow down the progression as shown above. Thus, combination of myopia control interventions is proposed as different mechanisms may be responsible for their effectiveness, adopting optical and pharmaceutical interventions at the same time may have additive or synergistic effects on myopia control. Our recent study demonstrated that a combination of orthokeratology and low dose atropine (0.01%) had additive effectiveness and reduced myopia progression by approximately 90% after one year of administration (17). Orthokeratology is one of the myopic control interventions that involves an overnight wear of rigid permeable lenses for reshaping the corneal profile (18). While the exact underlying mechanism of orthokeratology remains unknown (19), it is likely initiated by increasing peripheral myopic defocus after reshaping the cornea. The use of orthokeratology is widely accepted but is nevertheless associated with a risk of corneal infection due to the overnight lens wear. The compliance in dealing with orthokeratology might be more challenging when compared to spectacle lens wear because of the aftercare and disinfection of lenses. Moreover, the effect of overnight corneal reshaping declines during daytime due to corneal biomechanics, potentially limiting its effectiveness during the second half of the day. Apart from these considerations, the cost of orthokeratology is much higher than spectacle lenses so it may further hinder the compliance of combining orthokeratology and low dose atropine in myopia control.

Therefore, we propose combining a defocus incorporated multiple segments (DIMS) spectacle lens with low dose atropine to control myopia progression in schoolchildren as DIMS is a less invasive option than orthokeratology to control myopia and the compliance is believed to be higher as no disinfection of contact lenses is required. Unlike orthokeratology, DIMS applies a constant magnitude of myopic defocus (8). Moreover, low dose atropine (0.01%) is proposed to be administered twice per day in this project. Through this project, we aim to halt myopia progression in schoolchildren using the above-mentioned polytherapy.

# 5. Study Objective

The primary aim of this study is to investigate the effect of combination of optical defocus and low dose atropine in myopia control.

**6. Study Design**

The trial design is a 2-arm parallel group, interventional, randomized controlled, superiority trial.

## 6.1 Inclusion Criteria

Participants will be eligible if they are seven to twelve years of age and of Hong Kong Chinese descent. Refractive status requirements are myopia -0.75DS or more in SER in both eyes, astigmatism -1.50D or less in both eyes, anisometropia 1.50D or less in SER. Functional vision requirements are spectacles best corrected monocular VA 0.04 logMAR or better, normal colour vision and normal binocular function. Participants and their parents or guardians must sign and date the informed consent and assent form and they must be willing to comply with all study procedures and be available for the duration of the study. Participants will need to be willing to wear spectacle regularly and put on eye drops daily.

## 6.2 Exclusion Criteria

An individual who meets any of the following criteria will be excluded from participation in this study, including prior myopia control treatment including orthokeratology, defocus soft contact lenses, progressive addition lenses, bifocal lenses, myopia control lenses, atropine etc., previous or current participation in myopia control studies, presence of eye disease or binocular vision problems including strabismus, amblyopia, oculomotor nerve palsies, corneal diseases, myopic macular degeneration and posterior staphyloma etc., colour vision deficiency, prior ocular or corneal surgery, having any ocular and systemic diseases and abnormalities that may affect visual function, refractive development, having systemic disease that are contradictory to atropine, or known allergy to cyclopentolate hydrochloride or atropine.

## 6.3 Recruitment

Participants will be recruited from The University of Hong Kong (HKU Eye Centre and Grantham Hospital) and The Hong Kong Polytechnic University optometry clinic, advertising and promotion at primary and secondary schools. Potential participants will be identified through a search of current records, during routine clinical appointments and through public advertising. Initial contact will be conducted via Qualtrics questionnaire embedded in the email and the advertisement. A basic screening will be performed by the questionnaire to check for study eligibility if relevant. A follow-up telephone call or email will also be made by a researcher who will answer any questions related to the study.

Potential participants who meet the eligibility criteria from the screening questionnaire will be invited to have an eligibility examination. If the participant is a screen failure due to resolvable ocular conditions (grade one above corneal staining due to ocular dryness, chalazion, or allergic conjunctivitis), or intolerant to the puff of non-contact intraocular pressure measurement; these potential participants may be rescreened within two weeks. If there are improvements in these situations, rescreening participants should be assigned the same participant identifier as for the initial screening. Eligible participants and their parents will be given an information sheet. Contents and terms will be explained by the researcher. Participants and their parents or guardians will be required to sign assent and consent forms respectively if they agree to join the study.

As this study is a longitudinal study and the interventions require participants to wear spectacle lenses and use eye drops, special care will be taken to follow them. The participants will be asked to come back after one month of post-interventions as aftercare appointments to make sure there is no allergic response to atropine and the treatment interventions of the study are tolerable towards individuals. They will be reminded of their scheduled visits by phone calls and written information. Participants will also be asked to return for regular follow-up visits by the study ophthalmologist for dispensing atropine 0.01% eye drops.

## 6.4 Study Intervention

Eligible participants will be randomized to one of two groups, one group will receive low dose atropine (0.01%) plus DIMS spectacles (ATD Group); another one will receive low dose atropine (0.01%) plus single vision spectacles (AT Group). Participants will stay within these groups for 18 months.

### 6.4.1 Atropine

The 0.01% atropine eyedrops will be stored in single-use unit dose as a sterile topical ophthalmic solution and free of preservatives. The volume of each unit dose is 0.5ml in polyethylene plastic bottle. They will be provided to parents or guardians for administering the subjects as one drop in the morning and one drop at night before bedtime (twice a day) for 18 months. Each vial is disposed of daily to prevent microbial contamination. Participants and their guardian(s) will be instructed on proper protection against bright sunlight (sunglasses, hats) to avoid adverse effects resulting from atropine-dilated pupils. Scheduled ocular health assessment will be performed by an ophthalmologist after the dispensing of eye drops to the participants. An online log sheet will be given to record usage of the atropine eye drops.

### 6.4.2 Spectacles

At sixth- and twelfth-month follow-up visits, the criteria of requiring to update the prescription of the spectacle lenses for both groups are (any of these criteria): increase in myopia of 0.50D (spherical over-refraction) or more; habitual visual acuity equal to or worse than 6/9.

## 6.5 Randomization

Eligible participants will be randomly allocated in one-to-one ratio to the atropine only (AT) or atropine plus optical defocus (ATD) treatment groups using the block size of 4. AT group is denoted as A while ATD group is denoted as B. List of possible permutations are AABB, ABAB, ABBA, BABA, BAAB, BBAA. The allocation sequence will be generated by Excel by Principal investigator. The sequence will be stored in an online platform encrypted with password. The access to sequence will only be given to the site coordinator who is unmasked to assign the participants to the interventions.

## 6.6 Blinding

It is a single-blinded controlled clinical trial. Investigators, including ophthalmologists and optometrists, who are responsible for collection of data related to myopia progression will be blinded to study intervention. Masking subjects and their parents or guardians is not feasible since the lens design of the DIMS lenses and single vision lenses is easy to be detected. A data monitoring committee will be established to review adverse events during the study and ensure the safety of the study participants. He or she is also responsible for allocation of participants to different study groups. The masking procedures fulfil the CONSORT requirements for a single masked randomized clinical trial. The participants will be asked to wear the spectacles in full-time mode. A log sheet or an access to an online platform will be given to the participants, their parents or guardians for recording usage of eye drops. Compliance will be monitored and checked by phone calls and questionnaire.

## 6.7 Withdrawal Criteria

Participants (or parents / guardians for participants <18 years of age) are free to withdraw from participation in the study at any time upon request. No further data will be collected on participants who withdraw themselves from this study. If significant intolerance to study treatment is suspected at any time after randomization, participants may be requested to withdraw from the study at any time.

An investigator may discontinue or withdraw a participant from the study due to the following reasons:

- Significant study intervention noncompliance: unable to contact via three phone calls, more than three attempts to arrange missed follow-up appointments, wearing the spectacles less than five days a week and / or weekly average less than five hours/day.
- If any clinical adverse event or situation occurs (e.g. allergies to eye drops, reduction of visual function such as diplopia, reduction of BCVA; subject to clinicians’ judgement) such that continued participation in the study would not be in the best interest of the participant.
- If the participant meets an exclusion criterion (either newly developed or not previously recognized) that precludes further study participation.

Should participants require discontinuation of study treatment for any reason, or if they select to cease taking treatment, follow-up visits and data collection will not be continued as scheduled. The reason of discontinuation or withdrawal of participant from the study will be recorded on the Discontinuation / Withdrawal Case Report Form (CRF W) in Research Electronic Data Capture (REDCap). The use of REDCap can help reduce human errors in the randomization process, to assist data collection, and to provide a real-time dashboard for monitoring the project progress. Participants who sign the informed consent form, and are randomized but do not receive the study intervention may be replaced. If there are participants who signed the informed consent form, are randomized and received the study interventions but discontinue or withdraw within the first two weeks due to inability to meet inclusion criteria (e.g. unable to tolerate spectacles or eyedrops), and it is still within the recruitment period, then randomization of these participants will be discarded and replaced with a participant from the next allocation. All spectacles and eye drop (used or unused) will be returned to the investigator on withdrawal or discontinuation from the study.

## 6.8 Lost to Follow-up

A participant will be considered lost to follow-up if they fail to return for at least one of the data collection visits and is unable to be contacted by study staff. The following actions must be taken if a participant fails to return to the clinics for a required study visit:

- The investigators will attempt to contact the participant and reschedule the missed visit within 2 weeks of the original schedule and counsel the participant on the importance of maintaining the assigned visit schedule and ascertain if the participant and their family wishes to and/or should continue in the study.
- Before a participant is deemed lost to follow-up, the investigator will make every effort to regain contact with the participant (where possible, 3 telephone calls and, if necessary, a letter or an email to be sent to the participant’s last known mailing address or email). These contact attempts will be documented in the participants’ study file.
- Should the participant continue to be unreachable, they will be considered to have withdrawn from the study with a primary reason of lost to follow-up.

## 6.9 Eligibility and Baseline Assessment

All data will be collected via face-to-face meetings with researchers at The University of Hong Kong and Optometry Research Clinic of School of Optometry from The Hong Kong Polytechnic University. The following data will be collected at the baseline and eligibility assessment according to the manual of procedures.

- **Demographic data:** Ethnicity, age (date of birth) and gender.
- **Concomitant medication:** Information about types and dose of medications currently used (will be asked at all visits).
- **Allergies history:** Information about any allergies to medication or others (will be asked at all visits).
- **Health history:** Information about any previous ocular surgery and current or previous health problems related to vision or visual development.
- **Daily schedule:** Bedtime and wake up time.
- **Questionnaire:** Information about the history of myopia of the participants.
- **Visual acuity:** For best corrected VA, distance visual acuity will be measured using the highly standardized ETDRS letter chart adopted by the Amblyopia Treatment Study group (20), while habitual and subjective refraction VA will be measured by letters in Thomson Test Chart 2000 system. Near visual acuity will be measured using the LogMAR modified ETDRS near visual acuity letter chart.
- **Presence of strabismus:** Measured using the cover test.
- **Autorefraction and keratometry:** Measured using Shin-Nippon NVision-K 5001 open-field autorefractor. Five measurements will be taken and averaged only for autorefraction.
- **Pupil size:** Measured in photopic and mesopic lighting conditions using NeurOptics VIP-300 pupillometer.
- **Subjective refraction:** Measured by trial frame and trial lenses (maximum plus before blur) for sphere, Jackson cross cylinder for astigmatism and Thomson Test Chart 2000 system for VA.
- **Binocular vision with best subjective refraction:** Stereopsis is measured by Randot Stereotest, monocular and binocular amplitude of accommodation is measured by RAF rule, binocular accommodative lag is measured with 0D, 2D, 3D, 4D and 5D stimuli by Shin-Nippon NVision-K 5001 open-field autorefractor, and phoria is measured using Howell Phoria Card, or using cover test in the eligibility assessment. Five measurements will be taken and averaged only for binocular accommodative lag.
- **Colour vision**: Measured by Ishihara Test.
- **Intraocular pressure:** Measured by Topcon CT-80 Non-contact Computerised Tonometer.
- **Choroidal thickness:** Measured by Heidelberg Spectralis HRA+OCT with enhanced depth imaging mode (optical coherence tomography) or Topcon Triton Swept Source OCT.
- **External ocular health examination:** examined using a slit lamp biomicroscope.
- **Cycloplegia:** One drop of proxymetacaine 0.5% or Provain-POS 0.5% (topical anaesthetic) and 1 drop of cyclopentolate HCL 1.0% (antimuscarinic eye drop) will be instilled to both eyes to induce cycloplegia. Information sheet about drug will be distributed before performing cycloplegia, listing the drug name, effective ingredients, effective time, duration and side effects. The effect of the cycloplegia will be evaluated by means of amplitude of accommodation (push-up method) measured by RAF rule; if the amplitude of accommodation is more than +2.00D, then a second drop of cyclopentolate 1.0% will be instilled to both eyes. The remaining tests of this section will be performed under cycloplegia.
- **Cycloplegic autorefraction:** Same procedures as non-cycloplegic autorefraction. Five measurements will be taken and averaged.
- **Peripheral refraction:** Measured by Shin-Nippon NVision-K 5001 autorefractor at centre, 10˚, 20˚ and 30˚ of the nasal and temporal visual field across the horizontal meridian in both eyes. Five measurements will be taken and averaged.
- **Cycloplegic subjective refraction:** Same procedures as non-cycloplegic subjective refraction.
- **Axial length:** Measured by IOL Master 500 (Carl Zeiss) (partial coherence interferometry). Five measurements will be taken and averaged.
- **Ocular biometry:** Corneal curvature, corneal thickness, anterior chamber depth, lens thickness and axial length will be measured using Lenstar LS900 (optical low coherence reflectometry). Five measurements will be taken and averaged only for axial length.
- **Posterior ocular health examination:** examined using a binocular indirect ophthalmoscope and fundus photo (Topcon TRC-NW8 Fundus Camera).

## 6.10 Primary Outcome Measure

The primary outcome measures are changes in cycloplegic refraction in spherical equivalent and axial length over 18 months from baseline, as measured by cycloplegic autorefraction (Shin-Nippon open-field autorefractor) in both eyes and by IOL Master 500 (Carl Zeiss) respectively.

## 6.11 Secondary Outcome Measure

The secondary outcome measures are accommodative amplitude and response, pupil size, other ocular biometrics, which will be collected during first-, sixth-, twelfth- and 18^th^-month visits. Treatment compliance is checked by self-reporting log by participant at all the time points. Instillation of eye drop is checked by self-reporting log at all the time points. Information regarding to any adverse events or intervention events and whether they are related to the active treatment will be collected at all the time points.

All other measurements will be classified as exploratory outcomes.

## 6.12 Schedule of Intervention and Follow-up

| Procedures | Screening Questionnaire | Visit 0-1: Eligibility / Baseline | Visit 2: Spectacles and  Eye Drop Dispensing | Visit 3: First Month Aftercare | Visit 4-5: Sixth- and Twelfth-Month Follow-up | Visit 6: 18-Month Follow-up |
| --- | --- | --- | --- | --- | --- | --- |
| Informed Consent |  | X |  |  |  |  |
| Demographics | X | X |  |  |  |  |
| Medical History | X | X | X | X | X | X |
| Parental and Subject Refractive Status Questionnaire | X | X |  |  |  |  |
| Randomization |  | X |  |  |  |  |
| Administer Study Intervention |  |  | X | X | X |  |
| Visual Acuity (Habitual; D&N) |  | X | X | X | X | X |
| Cover Test |  | X |  |  | X | X |
| Interpupillary Distance |  | X |  |  | X | X |
| Non-cycloplegic Autorefraction and Keratometry |  | X |  |  | X | X |
| Non-cycloplegic Subjective Refraction and BCVA |  | X |  |  | X | X |
| Phoria |  | X |  |  | X | X |
| Anterior Ocular Health |  | X | X | X | X | X |
| NCIOP |  | X | X | X | X | X |
| Pupil Size |  | X | X | X | X | X |
| Stereopsis |  | X |  |  | X | X |
| Amplitude of Accommodation |  | X | X | X | X | X |
| Accommodative Lag |  | X |  |  | X | X |
| Contrast Sensitivity |  | X |  |  | X | X |
| Optical Coherence Tomography |  | X |  |  | X | X |
| Procedures | Screening Questionnaire | Visit 0-1: Eligibility / Baseline | Visit 2: Spectacles and  Eye Drop Dispensing | Visit 3: First Month Aftercare | Visit 4-5: Sixth- and Twelfth-Month Follow-up | Visit 6: 18-Month Follow-up |
| Cycloplegia |  | X |  |  | X | X |
| Cycloplegic Autorefraction |  | X |  |  | X | X |
| Peripheral Refraction |  | X |  |  | X | X |
| Cycloplegic Subjective Refraction |  | X |  |  | X | X |
| Axial Length |  | X |  |  | X | X |
| Ocular Biometry |  | X |  |  | X | X |
| Dilated Fundus Ophthalmoscopy |  | X |  |  | X | X |
| Order Spectacle Lenses |  | X |  |  | X | X |
| Verification of Spectacle Lenses |  |  | X | X | X | X |
| Verification of Atropine |  |  | X | X | X | X |
| Spectacle Lenses Dispensing |  |  | X |  | X | X |
| Atropine Dispensing |  |  | X | X | X | X |
| Visual Performance Questionnaire |  |  |  | X | X | X |
| Spectacle Wear and Eye Drop Log |  |  | X | X | X | X |
| Adverse Event Review and Evaluation | X | X | X | X | X | X |
| Complete Case Report Forms (CRFs) | X | X | X | X | X | X |

*Abbreviations: D&N Distance and Near; BCVA Best Corrected Visual Acuity; IOP Intraocular Pressure*

## 6.13 Data Collection Schedules

The following steps will be conducted by study personnel:

### 6.13.1 Eligibility, baseline assessments and randomization

- Screen potential participants through screening questionnaire and acquire contact details.
- Screen eligible participants through eligibility assessment (Case Report Form (CRF) in Research Electronic Data Capture (REDCap)).
- Perform baseline assessment on participants for eligibility (CRF Baseline in REDCap).
- Record refractive history (CRF RxHx in REDCap).
- Randomization of participants via REDCap (Randomization Log in REDCap).

### 6.13.2 Spectacles and atropine dispensing

- Order the type of spectacle lenses (Single vision or DIMS lenses) according to randomization (Randomization Log in REDCap) by an unmasked investigator.
- Order, record and dispense type of eye drops (atropine 0.01%) by an unmasked investigator (data recorded in CRF eyedrop in REDCap)
- Record results of delivery visual assessments (CRF Delivery in REDCap).

### 6.13.3 Follow-up assessments (data related to treatment outcomes)

The following steps will be completed by study personnel at the first-, sixth-, twelfth-, and 18-month follow-up appointments:

- Record results of follow-up visual assessments (CRF FU in REDCap).
- Record treatment compliance data (Treatment Compliance Log in REDCap).
- Record visual performance and treatment acceptability outcome data (Spectacle Vision and Comfort Questionnaire in REDCap).
- Record any adverse events affecting the participant and any incidental events (CRF AE in REDCap).
- Update spectacle lenses if required (CRF Delivery in REDCap).

## 6.14 Protocol Violations

A deviation from the protocol when no amendment has been submitted and approved would be regarded as a protocol violation. All protocol violations must be documented and reported (CRF V in REDCap). Prescribed treatment dose of spectacles is at least 70 hours each week (10 hours of spectacle wear per day, 7 days a week) and treatment dose of eye drop is two drops of eye drop every day, seven days a week. A participant is considered as compliant to the study protocol if he or she has worn the spectacle for at least 56 hours a week (7 days of at least 8 hours). If the spectacle lenses are worn for less than 35 hours per week, or eye drops are put on for less than three days per week, it is defined as a protocol violation. A list of such patients will be prepared prior to unblinding.

# 7. Statistical Considerations

## 7.1 Sample Size

In the study regarding low dose atropine for myopia control, mean change in refractive error after one year was 0.64 ± 0.56D (mean ± SD) (10). Our previous randomized clinical trial showed DIMS slowed down myopia progression by approximately 60% (8). The sample size is calculated based on an assumption that, adopting atropine and DIMS together will result in a 60% reduction of mean refractive error change relative to using only atropine, the difference detected between two groups (atropine alone vs. atropine and DIMS) will be 0.384 (0.64 x 60% = 0.384), and the effect size will be 0.685 (0.384/0.56 = 0.685). According to power analysis (G*Power Version 3.1.9.2), 46 subjects are required per group to achieve 90% power with a significance level of 0.05 (two-tailed). Assuming the dropout rate will be 20%, 56 subjects will be required for each group in this proposal. Therefore, 112 subjects (56 subjects × 2 groups) will be required for the entire study.

## 7.2 Statistical analysis

Statistical analyses will be performed using SPSS. Data of right eyes will be used for analyses. Data from the trial will be entered into an excel spreadsheet and extracted into a statistical program for analysis. Data analyses will be specified *a priori* in a statistical analysis plan (SAP) prepared by the researchers (and agreed upon by all members of the Steering Committee). All statistical tests will be two-tailed and a 5% significance level maintained throughout the analyses.

### 7.2.1 Baseline characteristics

Baseline demographics and clinical characteristics including ethnicity, age, gender, myopia onset, visual acuity, SER, wearing time of habitual spectacles on all randomised participants will be summarised using descriptive statistics.

### 7.2.2 Treatment effects

The effectiveness of myopic control will be calculated as:

1 – (progression therapy) / (progression control)

Unpaired t-test will be used to compare the changes in primary outcomes between two groups. If statistically significant differences in baseline demographic characteristics between groups are found, these significant covariates will also be adjusted in these primary analyses with Analysis of Covariates. In this case, both the unadjusted and adjusted results will be reported. Multiple regression analysis with the primary outcomes at the final visit will act as the dependent variable and independent variables, alongside with the secondary outcomes, will be employed to identify factors that may associate with myopia control effectiveness.

### 7.2.3 Tolerability

Treatment will be discontinued if any treatment-related adverse event arises, such as atropine allergy, constant visual disturbance (e.g. ghosting, diplopia, glare etc.). The number of participants discontinuing treatment prematurely for any reason will be summarised by treatment group and by reasons for discontinuation. The incidence of all serious adverse events will be summarised by treatment group.

### 7.2.4 Procedures to account for missing data

All analyses will use intention-to-treat analyses. Missing values of outcome variables and covariates will be replaced using multiple imputation procedure with 10 sets of imputations assuming missing at random. Sensitivity analyses for the primary outcomes include: complete case analysis and per-protocol analysis.

### 7.2.5 Interim analyses

No formal interim efficacy analyses of the outcome data are planned, as it seems unlikely that there would be sufficient data and reason to terminate the trial early.

## 7.3 Data management

### 7.3.1 Source data

The source documents are CRF entries. This includes CRFs available in Research Electronic Data Capture (REDCap) and an electronic spreadsheet designed according to the forms in REDCap.

REDCap is a web application for building and managing online databases (21). Data stored within REDCap, information pertaining to the identity and activities of REDCap users are well protected. REDCap is installed under the IT infrastructure and environment in The Hong Kong Polytechnic University (PolyU). Both the web server and database server are located behind the firewall of PolyU. Multi-factor authentication is required for the users to gain access to the REDCap. User will need to connect to a dedicated virtual private network using Microsoft multi-factor authentication pre-registered and installed in their smartphone, and then enter use a different set of login and password to login REDCap. Each user has their own account for the REDCap access to the project. Limited access to various functionality and modules such as being able to export data, to enter data or to modify the user privileges could be set up according to the role of investigators. Data Access Group could be implemented to segregate users between HKU and PolyU so the users will only gain access to necessary items so as to protect the privacy of the subject. Access to the REDCap will be limited to trial staff only. Anonymized patient identifiers will be used on all trial-specific documents, other than the signed consent forms and electronic folder of the participant (only available to the unmasked investigators). The participants will be referred to by four code initials, date of birth and registration numbers, not by name.

### 7.3.2 Data Recording and record keeping

Direct access will be granted to authorised representatives from the team members from The Hong Kong Polytechnic University and The University of Hong Kong. All trial data will be collected by research optometrists and administration personnel and recorded directly on to paper copies of the CRFs or CRFs in REDCap. Data will then be entered into the study spreadsheets by authorised personnel at each site. Data will be backed-up onto a secure central cloud server. Only authorised, trained personnel will have access to the electronic data. The participants will be identified by a unique trial specific number and/or code in the spreadsheet. The name and any other identifying detail will not be included in any electronic files.

# 8. Ethical Approval and Consent

## 8.1 Ethics Approval

The researchers will seek ethical approval from Institutional Review Board of the University of Hong Kong/Hospital Authority Hong Kong West Cluster (HKWC/HKU Cluster IRB), and The Institutional Review Board of the Hong Kong Polytechnic University, prior to recruiting participants to the study.

## 8.2 Informed Consent

All participants will be given a copy of their written consent details and a participant information sheet. The consent form will be signed by both the participant, their parents/ guardians and an authorised researcher at the beginning of the first clinic visit. All data collected will be treated as confidential and electronic data will be stored securely at the Hong Kong Polytechnic University. The trial staff will ensure that the participants’ anonymity is maintained.

# 9. Assessment of Safety/Adverse Event Reporting

## 9.1 Adverse Events

### 9.1.1 Definition of an Adverse Event

Adverse event (AE) means any unfavourable and unintended sign including any abnormal laboratory finding, symptom or disease associated with the use of an intervention in humans, whether or not considered intervention-related, during the course of the study. Any incident associated with the eye drop will be recorded and reported through the same system as an AE (CRF AE in REDCap).

### 9.1.2 Contact for Notification of Adverse Events

Worsening of visual acuity, development of visual disturbances (such as diplopia, ghosting, glare), eye strain symptoms, symptoms of eye infection or allergic responses (such as redness, pain, decreased vision, discharge, increased light sensitivity) are to be noted on the Case Report Form (CRF AE) in REDCap. The relationship of each adverse event to the trial treatment must be determined by a clinically qualified individual according to the following definitions:

- **Related:** The adverse event follows a reasonable temporal sequence from trial treatment. It cannot reasonably be attributed to any other cause.
- **Not Related:** The adverse event is probably produced by the participant’s clinical state, by other modes of therapy administered to the participant or is otherwise unrelated to the trial treatment.

All AEs and other incidents occurring during the trial that are observed by study personnel or reported by the participant will be recorded on the CRF in REDCap, whether or not attributed to trial treatment. The following information will be recorded: description, date of onset and end date, severity, assessment of relatedness to trial intervention, other suspect drug or device and action taken. Follow-up information should be provided as necessary. The severity of events will be assessed on the following scale:

- **Mild (1):** Events require minimal or no treatment and do not interfere with the participant’s daily activities.
- **Moderate (2):** Events result in a low level of inconvenience or concern with the therapeutic measures. Moderate events may cause some interference with functioning.
- **Severe (3):** Events interrupt a participant’s usual daily activity and may require systemic drug therapy or other treatment. Severe events are usually potentially life-threatening or incapacitating. Of note, the term “severe” does not necessarily equate to “serious”.

These data on adverse event reports will be tabulated in a spreadsheet and reviewed quarterly at the Study Management Committee meetings.

Information to be collected includes event description, time of onset, clinician’s assessment of severity, relationship to study product (assessed only by those with the training and authority to make a diagnosis), and time of resolution or stabilization of the event. All AEs occurring while on study must be documented appropriately regardless of relationship. All AEs will be followed to adequate resolution. An appointment will be arranged for four to six weeks following AE resolution to check for any recurrence or changes as a result of the AE. Changes in the severity of an AE will be documented to allow an assessment of the duration of the event at each level of severity to be performed. AEs characterized as intermittent require documentation of onset and duration of each episode. These will be followed-up and assessed by medically fit clinician. It will be left to the judgement of the Steering Group to decide whether or not an AE is of sufficient severity to require discontinuing the participant from the study. A participant may also voluntarily withdraw from participating in the study due to what he or she perceives as an intolerable AE. If either of these occurs, the participant must undergo follow-up visits for trial assessment and be given appropriate care under medical supervision until symptoms cease, or the condition becomes stable.

## 9.2 Serious Adverse Events

### 9.2.1 Definition of a Serious Adverse Event

A serious adverse event (SAE) is any untoward medical occurrence that

- results in death, or
- is life-threatening (defined as an event in which the participant was at risk of death at the time of the event; it does not refer to an event which hypothetically might have caused death if it were more severe.), or
- requires inpatient hospitalization or prolongation of hospitalization, or
- results in persistent or significant disability or incapacity, or
- requires intervention to prevent permanent damage (the use of the test article resulting in a condition which requires medical or surgical intervention to preclude permanent impairment of the body structure or a body function). Important medical events that may not result in death, be life-threatening, or require hospitalization may be considered a serious adverse event when, based upon appropriate medical judgment, they may jeopardize the patient or subject and may require medical or surgical intervention to prevent one of the outcomes listed in the above definition.

Examples of such medical events include:

- Microbial Keratitis (MK).
- Permanent decrease in best spectacle corrected visual acuity equivalent to two acuity lines or greater.
- Anaphylactic shock from hypersensitivity to eye drops or test article.

### 9.2.2 Significant Adverse Events

Events that are usually symptomatic and warrant discontinuation (temporary or permanent) of the test article (excluding Serious Adverse Events).

Diagnoses and conditions that are considered Ocular Significant Adverse Events include, but not limited to the following:

- Any Temporary Loss of > two Lines of BCVA.
- Other grade three or higher corneal findings, such as abrasions or oedema.
- Non-contact lens related corneal events - e.g. Epidemic Keratoconjunctivitis (EKC).
- Asymptomatic Corneal Scar.
- Intermittent Spiking of Intraocular Pressure.
- Any corneal event which necessitates temporary eyedrop discontinuation > two weeks.

### 9.2.3 Non-Significant Adverse Events

Conditions that are usually asymptomatic and usually do not warrant discontinuation (temporary or permanent) of the test article. However, the investigator may choose to treat as a precautionary measure.

Diagnoses and conditions that are considered Ocular Non-Significant Adverse Events include, but not limited to the following:

- Conjunctivitis: Bacterial, Viral, Allergic
- Blepharitis
- Meibomianitis
- Contact Dermatitis
- Localized Allergic Reactions
- Any corneal event not explicitly defined as serious or significant adverse event, which necessitates temporary eye drops discontinuation < two weeks.

### 9.2.4 Contact for notification of serious adverse events

All SAE will be recorded on an adverse event form (CRF AE) in REDCap. If known, the name of the underlying diagnosis will be recorded, rather than its individual symptoms. The data on CRF AE will be entered into the study spreadsheet and must be reported through email attachment expeditiously to the Study Management Committee for notification within 72 hours of the study team becoming aware of the event. The Study Management Committee will perform an initial check of the report and request any additional information. Follow-up checks will be on a weekly basis. The event will be reviewed at the next Study Management Committee meeting. All SAE information must be recorded, scanned and emailed to Steering Group Committee Members and Study Management Committee members.

## 9.3 Reporting safety information

The project coordinator will submit updated safety information to the Steering Group Committee and Study Management Committee every three months and will submit updated safety information throughout the clinical trial every year, or on request, to the approval authorities stated in section 7.1.

## 9.4 Unblinding

This is a single-blinded trial, where research clinical examiners responsible for data collection are blinded to treatment allocation. Unblinding will only occur at the end of the study under the authorization of the project coordinator.

For individual emergency unblinding, where a serious adverse event has occurred and the group allocation of the individual is required in order to enable clinical treatments to be planned, project coordinator will be informed of the allocation of the individual and this will be documented. The masked research clinical examiner will remain blinded.

## 9.5 Data safety and monitoring

Ellenburg et al. (22) provide guidelines for deciding whether or not a data safety monitoring committee (DSMC) needs to be established for a trial . They proposed that if two or more of the following criteria are met, then a DSMC is required:

- The trial is intended to provide definitive information about the effectiveness and / or safety of a medical intervention.
- There is prior data to suggest that the intervention being studied has the potential to induce potentially unacceptable toxicity.
- The trial is evaluating mortality or another major endpoint such that inferiority of one treatment group has safety as well as effectiveness implications.
- It would be ethically important for the trial to stop early if the primary question addressed has been definitively answered, even if secondary questions or complete safety information were not fully addressed.

This trial meets only the first point indicating that an independent DSMC does not need to be established for the trial. However, data safety and regular monitoring will be performed by steering group committee and study management committee. Compliance with the protocol and accuracy in relation to source documents will be evaluated.

An independent monitor will check the existence and correct date for all signed consent forms. The monitor will sample over 10% of all randomized participant to check correct data collection and data entry for the key points of this study, including visual acuity and treatment / control placement. The monitor will confirm collected data on CRFs in REDCap with the source data and check the data entry from the hard copies such as print out data from open-field autorefractor.

# 10. Intervention supplies

## 10.1 Study treatment identification

The following products will be used for intervention in this study:

| Products | Supplier |
| --- | --- |
| DIMS spectacle lenses | HOYA Lens Hong Kong Limited |
| Single Vision spectacle lenses | HOYA Lens Hong Kong Limited or equivalent |
| Atropine 0.01% | Aseptic Innovative Medicine Co. Ltd,  Taiwan or equivalent |

## 10.2 Handing and Dispensing of Study Treatment

All spectacles and eye drops used in this study are maintained under the direct responsibility of the project coordinator. It will be the responsibility of project coordinator to ensure that an accurate record of the interventions issued to participants is maintained. All spectacles, whether worn or not worn, and unused eye drops will be returned to the project coordinator at the end of the study or on withdrawal / discontinuation from the study.

## 10.3 Packaging and Labelling

### 10.3.1 Spectacle Lenses

When the spectacle lenses arrive from the supplier, these will be recorded in an inventory log by the unmasked investigator. Verification and relabeling of the lenses with the unique participant identifier to ensure blinding will be organized by the unmasked investigator.

### 10.3.2 Atropine

When the atropine 0.01% eye drops arrive from the supplier, these will be recorded in an inventory log by the unmasked investigator. Verification and relabeling of the eye drops with the unique participant identifier according to the allocation to ensure blinding will be organized by the unmasked investigator.

## 10.4 Treatment Supply Records

It will be the responsibility of the project coordinator to ensure that an inventory of the interventions is maintained. Records or logs will include:

- Amount and record of devices and eye drops used in the study.
- Dates of device and eye drops inventory movement.
- Unique participant identifier (when allocated).
- Type of intervention dispensed to each participant.
- Initials of the person who dispensed the interventions.

Intervention dispensing and recording will be completed by unmasked personnel in the study. A set template using an Excel spreadsheet will be used to record this process at the study site.

# 11. Relevance to Health

In young children in Hong Kong and other parts of East Asia, annual progression of myopia can be as rapid as 1.00D or more. A highly effective treatment will proportionally lower the final amount of myopia when he / she reaches adulthood. It is believed the earlier the treatment is applied, the lower the amount of final myopia will be. Our ultimate goal is to develop methods that slow myopia progression by 90% or higher, preferably using a minimally invasive approach applicable to younger children as early as age of six. Our hypothesis is that combining interventions will increase the effectiveness, so that the prevalence of adult high myopia (> 5D) and the associated visual impairment can be dramatically reduced. The current proposal is a two-arm clinical trial. We aim to answer whether combination treatments involving combinations of myopic defocus and low dose atropine will be more effective than monotherapy in controlling myopia of schoolchildren.

The short- and medium-term beneficiaries of myopia control are myopic schoolchildren. The results can directly benefit schoolchildren in terms of providing the best treatment strategy or strategies to slow their myopia progression. This is particular important in the early school years when myopic children rapidly progress towards high myopia. A large number of children can benefit from the prevention of progression to high myopia after the size of their eye stabilize at around 20 years old. There are three aspects of long-term beneficiaries from the prevention of high myopia: individual level, public health care system and the society: successful myopia control for individuals can benefit them over their lifetime, as they will not suffer from the associated visual loss and blindness. Individuals with high myopia otherwise would have an increased risk of sight-threatening ocular diseases, such as glaucoma, retinal detachment, myopic maculopathy, and premature cataracts which can permanently impair their vision. With an effective myopia progression treatment, they could maintain good quality of life without vision impairments which would otherwise restrict mobility and impact daily living. In addition, they will avoid the social economic burden due to decreased productivity and cost of long-term care for these ocular complications and blindness associated with high myopia.

The Public Health care system is the second beneficiary. Without a successful intervention, the increased prevalence of myopia in the aging myopic population will impose an excessive burden on already stressed public eye care services. The prevention of high myopia would reduce the number of people suffering from myopia related vision impairments and therefore alleviate the burden to the public health care system. The society is also a beneficiary. The total costs of myopia to the society have not been comprehensively estimated. It includes myopia corrections, public ophthalmologic services, training extra optometrists and ophthalmologists to cope with the increased myopia prevalence, social welfare costs for the visually impaired, cost of accident e.g. falls due to visual impairment, as well as productivity loss due to vision impairments.

The proposed project aims to develop an optimal protocol for controlling of myopia in a clinical setting. It will provide clinicians with new directions and tools, providing vital information for clinical decision making. It will also provide new opportunities for industrial partners to commercialize these methods for myopia control and benefit the community at large.

# 12. Dissemination of Results

## 12.1 Trial Registration

The trial will be registered on Clinicaltrials.gov before starting subject recruitment and randomization.

## 12.2 Study Participants

At the completion of participation, oral information will be given on the treatment effects of this myopia control intervention. The participant and family will be informed of the myopia progression rate for the participant from the trial and their last prescription. All the participants will receive a letter of thanks for participating in the study, a brief summary of the study results and an outline of their significance when the study is complete.

## 12.3 Academic / Professional Colleagues

Articles detailing the trial results will be submitted to leading international peer-reviewed journals and data will be presented to international audiences and conferences.

# 13. Administrative Section

## 13.1 Adherence to Protocol

The approved protocol will be strictly followed throughout the trial. Exceptions will only apply to eliminating an immediate hazard to participants. Any protocol deviation will be documented in the protocol violations form (CRF V).

## 13.2 Protocol Revision Procedures

All revisions will be discussed and approved by the Study Steering Committee. Any amendment will be submitted by the project coordinator to the Institutional Review Board of the Hong Kong Polytechnic University and The University of Hong Kong for review and approval or favourable opinion prior to implementation. Documentation of approval signed by the chairperson or designee of the Institutional Review Board and sent to the project coordinator will be filed.

Procedures listed below will be followed if an amendment substantially alters study design or increases the potential risk to the subject:

- The consent form will be revised and submitted to the Institutional Review Board for review and approval or favourable opinion;
- If current enrolled participants are affected by the amendment, the participants will firstly be contacted by telephone, the amendment will be discussed and verbal consent will be re-obtained;
- Furthermore, the revised consent form will be sent to current enrolled participants by post.

## 13.3 Case report form procedures

All information will be collected onto CRFs in REDCap. For all CRFs, participants will be identified by initials, date of birth and registration number, except CRF Baseline, which contains participant’s identifiable details. CRF Baseline will be locked with other identifiable material and this information will not be entered into the study spreadsheet or released from the study centre. All requested information on the CRF will be entered in the spaces provided. Blank spaces will not be permitted. An asterisk ‘*’ will be entered if the data is unavailable and a dash ‘-’ will be entered if the data is not applicable.

## 13.4 Monitoring / Source Document Verification

Data collection and data entry onto the study spreadsheet will be monitored closely and regularly. The monitor will review all registered participants’ records to ensure that they have provided informed consent. The monitor will review the study documentation and records to ensure that all documentation is up-to-date, including correct version of the Study Protocol and Manual of Procedures and the record-keeping meets the requirements specified in the protocol.

The monitor will also audit that the product supply records are maintained and that there are sufficient supplies remaining. The handling process will also be monitored against the study procedure.

## 13.5 Data Confidentiality and Security

All CRF datasheets and consent forms collected from the participants will be treated as confidential and stored securely at each study site. The secure cloud server and participant records will be accessed only by the researchers involved in the study. REDCap employs various method to protect against malicious users who attempts to identify and exploit the security vulnerabilities in the REDCap. Processes such as sanitization, filtering, data type checking and escaping are implemented to help protect against methods of attack. Names of participants will not be included in published data. All the participants will only be identified by code when data is sent outside of the institution.

## 13.6 Reporting Schedule

The project coordinator will provide annual reports of study progress to the approving authorities stated in section 7.1. All serious adverse events that are fatal or life-threatening and suspected of being related to the treatment used in the trial will be reported to the local ethics committee within 72 hours.

## 13.7 Record Retention Policy

The source documents / data, including paper and electronic files, will be kept for 7 years from the date of study termination. Staff involved in the study will not destroy any record associated with the trial without the prior approval from the project coordinator.

If any co-investigators withdraw from the study (e.g. relocation or retirement), any records they hold will be transferred to a mutually agreed upon designee, such as another co-investigator. Transfer mentioned above will be noticed in writing.

## 13.8 Insurance

Participants in this trial are covered under the Hong Kong Polytechnic University “Blanket Clinical Trial Insurance” policy.

## 13.9 Ownership of Data and Publication Policy

Individual study data will remain the property of individual study participants. The Steering Committee will have responsibility for the safe guardianship and use of the data. All publications will be approved by members of the Steering Committee, who will be named on all papers and presentations. Study participants, the research clinicians, members of the Management Committee who are not part of the Steering Committee, and the study sponsor will be acknowledged in all papers and in all presentations resulting from this trial.

## 13.10 Declaration of interests

The principal investigator and the team members declare no competing interests.

# 14. Abbreviations

| AE | Adverse event |
| --- | --- |
| AL | Axial length |
| BC | Base curve |
| BCVA | Best corrected visual acuity |
| Co-I | Coinvestigator |
| CRF | Case report form |
| D | Dioptres |
| DIA | Diameter |
| DIMS | Defocus Incorporated Multiple Segments |
| DSMC | Data safety monitoring |
| E-ETDRS | Electronic Early Treatment Diabetic Retinopathy Study (chart) |
| FDA | Food and drug administration (United States of America) |
| HD | Hyperopic defocus |
| HSESC | Human Subjects Ethics Sub-Committee |
| ITT | Intention to treat |
| MD | Myopic defocus |
| MI | Masked investigator |
| MK | Microbial keratitis |
| MOP | Manual of procedures |
| NCIOP | Non-contact intra-ocular pressure |
| IOP | Intra-ocular pressure |
| IPD | Interpupillary distance |
| OCT | Optical coherence tomography |
| PI | Principal investigator |
| PolyU | The Hong Kong Polytechnic University |
| PR | Peripheral refraction |
| RCT | Randomised controlled trial |
| RPR | Relative peripheral refraction |
| Rx | Refractive error |
| SAE | Serious adverse event |
| SAP | Statistical analysis plan |
| SER | Spherical equivalent refraction |
| SD | Standard deviation |
| SV | Single vision |
| VA | Visual acuity |
| UMI | Unmasked investigator |
| 95% CI | 95% confidence interval |

# 15. References

1. Morgan IG, Ohno-Matsui K, Saw SM. Myopia. The Lancet (British edition). 2012;379(9827):1739-48.

2. Lam CSY, Lam CH, Cheng SCK, Chan LYL. Prevalence of myopia among Hong Kong Chinese schoolchildren: changes over two decades. Ophthalmic & physiological optics. 2012;32(1):17-24.

3. Holden BA, Fricke TR, Wilson DA, Jong M, Naidoo KS, Sankaridurg P, et al. Global Prevalence of Myopia and High Myopia and Temporal Trends from 2000 through 2050. Ophthalmology (Rochester, Minn). 2016;123(5):1036-42.

4. Mitchell P, Hourihan F, Sandbach J, Wang JJ. The relationship between glaucoma and myopia: The blue mountains eye study. Ophthalmology (Rochester, Minn). 1999;106(10):2010-5.

5. Saw SM, Gazzard G, Chan SYE, Chua WH. Myopia and associated pathological complications. Ophthalmic & physiological optics. 2005;25(5):381-91.

6. Zheng F, Wong CW, Sabanayagam C, Cheung YB, Matsumura S, Chua J, et al. Prevalence, risk factors and impact of posterior staphyloma diagnosed from wide‐field optical coherence tomography in Singapore adults with high myopia. Acta ophthalmologica (Oxford, England). 2021;99(2):e144-e53.

7. Jonas JB, Ang M, Cho P, Guggenheim JA, He MG, Jong M, et al. IMI prevention of myopia and its progression. Investigative ophthalmology & visual science. 2021;62(5):6-.

8. Lam CSY, Tang WC, Tse DYY, Lee RPK, Chun RKM, Hasegawa K, et al. Defocus Incorporated Multiple Segments (DIMS) spectacle lenses slow myopia progression : a 2-year randomised clinical trial. British journal of ophthalmology. 2020.

9. Tong LFDM, Huang XLB, Koh ALTB, Zhang XM, Tan DTHFF, Chua W-HFF. Atropine for the Treatment of Childhood Myopia: Effect on Myopia Progression after Cessation of Atropine. Ophthalmology (Rochester, Minn). 2009;116(3):572-9.

10. Chia AF, Chua W-HFF, Cheung Y-BP, Wong W-LM, Lingham ASRN, Fong AF, et al. Atropine for the Treatment of Childhood Myopia: Safety and Efficacy of 0.5%, 0.1%, and 0.01% Doses (Atropine for the Treatment of Myopia 2). Ophthalmology (Rochester, Minn). 2012;119(2):347-54.

11. Yam JC, Jiang Y, Tang SM, Law AKP, Chan JJ, Wong E, et al. Low-Concentration Atropine for Myopia Progression (LAMP) Study: A Randomized, Double-Blinded, Placebo-Controlled Trial of 0.05%, 0.025%, and 0.01% Atropine Eye Drops in Myopia Control. Ophthalmology (Rochester, Minn). 2019;126(1):113-24.

12. Li FF, Zhang Y, Zhang X, Yip BHK, Tang SM, Kam KW, et al. Age Effect on Treatment Responses to 0.05%, 0.025%, and 0.01% Atropine. Ophthalmology (Rochester, Minn). 2021;128(8):1180-7.

13. Zheng YF, Pan CW, Chay J, Wong TY, Finkelstein E, Saw SM. The economic cost of myopia in adults aged over 40 years in Singapore. Investigative ophthalmology & visual science. 2013;54(12):7532-7.

14. Gan J, Li S-M, Wu S, Cao K, Ma D, He X, et al. Varying Dose of Atropine in Slowing Myopia Progression in Children Over Different Follow-Up Periods by Meta-Analysis. Frontiers in medicine. 2022;8:756398-.

15. Wildsoet CF, Chia A, Cho P, Guggenheim JA, Polling JR, Read S, et al. IMI – Interventions myopia institute: Interventions for controlling myopia onset and progression report. Investigative ophthalmology & visual science. 2019;60(3):M106-M31.

16. Schaeffel F, Feldkaemper M. Animal models in myopia research: Animal models in myopia researchSchaeffel and Feldkaemper. Clinical and experimental optometry. 2015;98(6):507-17.

17. Tan Q, Ng ALK, Choy BNK, Cheng GPM, Woo VCP, Cho P. One‐year results of 0.01% atropine with orthokeratology (AOK) study: a randomised clinical trial. Ophthalmic & physiological optics. 2020;40(5):557-66.

18. Hiraoka T. Myopia Control With Orthokeratology: A Review. Eye & contact lens. 2022;48(3):100-4.

19. Li X, Friedman IB, Medow NB, Zhang C. Update on orthokeratology in managing progressive myopia in children: Efficacy, mechanisms, and concerns. Journal of pediatric ophthalmology and strabismus. 2017;54(3):142-8.

20. Beck RW, Moke PS, Turpin AH, Ferris FL, SanGiovanni JP, Johnson CA, et al. A computerized method of visual acuity testing: Adaptation of the early treatment of diabetic retinopathy study testing protocol. American journal of ophthalmology. 2003;135(2):194-205.

21. Harris PA, Taylor R, Thielke R, Payne J, Gonzalez N, Conde JG. Research electronic data capture (REDCap)—A metadata-driven methodology and workflow process for providing translational research informatics support. Journal of biomedical informatics. 2009;42(2):377-81.

22. Ellenberg SS, Fleming TR, DeMets DL. Data monitoring committees in clinical trials a practical perspective. Chichester ;: Wiley; 2002.

# 16 Appendices

**Information Sheet**

**Project Title**

Combination Effect of Optical Defocus and Low Dose Atropine in Myopia Control – a Randomized Clinical Trial

**Research Team**

Dr. Ka Man CHUN, School of Optometry, The Hong Kong Polytechnic University (PolyU)

Prof. Chi Ho TO, School of Optometry, PolyU

Prof. Christopher Kai Shun LEUNG, Department of Ophthalmology, The University of Hong Kong

Dr. Yan Yin TSE, School of Optometry, PolyU

Dr. Sheung Shun NG, School of Optometry, PolyU

**Why is the study being performed?**

The aim of this study is to investigate the effect of combination of optical defocus and low dose atropine in myopia control. Optical defocus is characterized as myopic defocus and is generated by optical lenses to form images in front of the eyes. Both myopic defocus and low dose atropine have been applied for controlling myopia progression in children.

**Inclusion Criteria**

We recruit schoolchildren with the following inclusion criteria.

- Age at enrolment: 7 - 12 years
- Ethnicity: Hong Kong Chinese
- Myopia: -0.75DS (in spherical equivalent) or more in both eyes
- Astigmatism: -1.50DC or less in both eyes
- Difference in refraction between two eyes: 1.50D or less (in spherical equivalent)
- Best corrected monocular visual acuity (VA): 0.04 logMAR or better
- Ocular health: No abnormalities in both internal and external ocular health
- Systemic health: No abnormalities such as cardiac and respiratory diseases
- Binocular vision: No strabismus, diplopia, suppression and other binocular abnormalities
- Normal colour vision
- No previous refractive surgery or use of myopic control interventions, such as atropine, orthokeratology, and specialized spectacle lenses and contact lenses for myopic control
- Able to wear the prescribed spectacles
- No known allergy to atropine

**What do volunteers for the study have to do?**

If you and your child volunteer for the study, you and your child will be asked:

1. to sign an informed consent/assent form that states you and your child understand the information presented on this sheet

2. to provide your child’s name, age and sex

In the study, single-use unit dose, sterile and unpreserved 0.01% atropine eye drops will be provided to your child. The eyedrops will be prescribed and given by an ophthalmologist in HKU Eye Centre of The University of Hong Kong. Your child will also receive a pair of spectacles with either Defocused Incorporated Multiple Segments (DIMS) lens or single vision powered lens. Your child is required to use atropine eye drop twice per day (one drop in the morning and one drop at night before bedtime) and wear our spectacles most of the time during the day and an adaptation period is needed for the spectacles. In order to ensure the compliance, you/ your child is required to report the time and use of atropine eye drop daily to an online system. Your child has to return all used and unused eyedrops in the follow-up visits. Your child will be required to have follow up visits in HKU Eye Centre of The University of Hong Kong or Optometry Clinic of The Hong Kong Polytechnic University after 1 month of treatment and at 6-month for the first half year and then every 6 months for the rest of study period.

The duration of the study lasts for 18 months. The examination includes visual acuity, colour vision, refraction and ocular health assessment and it takes around 2 hours. You and your child will be benefited from regular follow up on myopia progression. We will also provide the frame and lenses for regular replacement. Refraction will be checked with the help of eye drops (Alcaine 0.5% or Provain-POS 0.5% plus cyclopentolate 1%) so as to relax the focusing power of the eyes. Mild stinging sensation will be experienced for few seconds when the eye drop is instilled. Your child will have blur vision at near and be sensitive to bright light for about 24 hours after the eye drops. He/ she is suggested to wear sunglasses or have a hat when having outside activities during the above period. Potential risks are listed on a separate information sheet of eye drops.

Measurements will be taken by non-contact ophthalmic devices and each measurement would be done within several minutes. It is hoped that this information will help understand the effect of myopia control by DIMS lenses and atropine eye drops.

**Schedule of Visits**

Your child is required to attend 6 visits (Table 1) during the study. In the 2^nd^ to 6^th^ visits, you and your child are required to go to both The Hong Kong Polytechnic University and The University of Hong Kong in order to perform eye examination and receive atropine eye drop, respectively.

Table 1. Schedule of visit

| Visit | Time | Purpose |
| --- | --- | --- |
| 1 | --- | Screening plus baseline measurement (PolyU) |
| 2 | ~ 1 month after baseline | Dispensing of spectacle (PolyU) and eyedrops (HKU) |
| 3 | 1st month after delivery | Follow up assessment in PolyU and HKU |
| 4 | 6th month | Follow up assessment in PolyU and HKU |
| 5 | 12th month | Follow up assessment in PolyU and HKU |
| 6 | 18th month | Follow up assessment in PolyU and HKU |

**Potential Risks**

The testing should not result in any undue discomfort. Mild stinging sensation will be experienced for few seconds when the eye drop for refraction or atropine for treatment in the study is instilled to your child. Your child will have blur vision at near and be sensitive to bright light for about 24 hours after the eye drops. He/ she is suggested to wear sunglasses or have a hat when having outside activities. Potential risks are listed in the information sheet of eye drops. Potential risks of spectacles such as blurriness, dizziness and interrupted binocular vision may be involved after wearing the new spectacles. These adaptational problems will usually ease off after few days of spectacles wear. You should report to the investigators if such problems happen via phone call (Dr Ka Man CHUN, Tel: 27664224 or 96590580).

**Benefits**

The refractive errors and ocular condition of your child will be followed up for 18 months. The myopia progression might be slowed down with the combination of myopic control interventions.

**Alternative treatment if child opts for not joining the study**

Refusal to participate or withdrawal at any time will not affect the child’s present or future normal medical care. Withdrawal children will be given the standard routine clinical management, which does not include the aforementioned additional follow-up visits and examinations.

**Can a volunteer withdraw from the study?**

Yes, you can stop participating in the study at any time with no penalty.

**Circumstances under which your participation in the Research will be terminated**

We reserve the right to terminate your participation in the research project. In the event that any safety concerns are raised during the study or your child are not able to attend follow up visits and compete the examinations, your participation will no longer be required.

**Information Confidentiality**

Any information that is obtained in this study concerning you will be confidential. The retention period of the research records will be at least 7 years. Publication or other public distribution of the experimental results will not mention your child by name. Raw data with your personal information will be destroyed upon the completion of this project. Research records will be stored securely and only researchers will have access to the records. The Institutional Review Board of The Hong Kong Polytechnic University and Institutional Review Board of the University of Hong Kong / Hospital Authority Hong Kong West Cluster will also have access to records in relation to ethics review purposes.

You and your child have the right of access to personal data and publicly available study results, if and when needed. Under the laws of Hong Kong (in particular the Personal Data (Privacy) Ordinance, Cap 486), you and your child enjoy or may enjoy rights for the protection of the confidentiality of your personal data, such as those regarding the collection, custody, retention, management, control, use (including analysis or comparison), transfer in or out of Hong Kong, non-disclosure, erasure and/or in any way dealing with or disposing of any of your personal data in or for this study. For any query, you should consult the Privacy Commissioner for Personal Data or his office (Tel No. 2827 2827) as to the proper monitoring or supervision of your personal data protection so that your full awareness and understanding of the significance of compliance with the law governing privacy data is assured.

By consenting to participate in this study, you expressly authorize:

• the principal investigator and her research team and the ethics committee (Institutional Review Board of The Hong Kong Polytechnic University and The University of Hong Kong / Hospital Authority Hong Kong West Cluster) responsible for overseeing this study to get access to, to use, and to retain your personal data for the purposes and in the manner described in this informed consent process; and

• the relevant government agencies (e.g. the Hong Kong Department of Health) to get access to your personal data for the purposes of checking and verifying the integrity of study data and assessing compliance with the study protocol and relevant requirements.

**Can I get more information on the study?**

Yes, please you may ask our researcher now or even after the study has started.

You may also contact Dr Ka Man CHUN (Tel. no.: 27664224 or 96590580 / email: rachel.chun@polyu.edu.hk) of The Hong Kong Polytechnic University or Professor Christopher Leung (HKU) at Tel: 2518 1430 under the following situations:

a. if you and your child have any other questions in relation to the study;

b. if, under very rare conditions, your child become injured as a result of your participation in the study; or

c. if you want to get access to / or change your personal data before 30 Nov 2026.

This study has been approved by the ethics committee (Institutional Review Board of the University of Hong Kong / Hospital Authority Hong Kong West Cluster) and Institutional Review Board of the Hong Kong Polytechnic University.

The study will be conducted in accordance with the ethical principles that have their origin in the Declaration of Helsinki, and that are consistent with GCP and the applicable regulatory requirement(s).

For enquiries concerning ethical right, please contact the Institutional Review Board of the University of Hong Kong / Hospital Authority Hong Kong West Cluster by phone (Tel: 2255 4086).

If you have any complaints about conduct of this study, you may contact Secretary, PolyU Institutional Review Board (institutional.review.board@polyu.edu.hk) stating clearly the responsible person and department of this study as well as the Reference Number (HSEARS20221122002).

Thank you for your interest in participating in this study.

Dr Ka Man CHUN

Principal Investigator

**Research Project Informed Consent Form**

**Project Title**

Combination Effect of Optical Defocus and Low Dose Atropine in Myopia Control – a Randomized Clinical Trial

**Research Team**

Dr. Ka Man CHUN, Prof. Chi Ho TO, Prof. Christopher Kai Shun LEUNG, Dr. Yan Yin TSE, Dr. Sheung Shun NG

| 1. Consent of the Parent/Guardian |  |
| --- | --- |
| Have you read the information sheet provided? | Yes / No |
| Have you had any opportunity to ask questions and discuss about this study? | Yes / No |
| Have you received satisfactory answers to all of your questions? | Yes / No |
| Have you received enough information about the study? | Yes / No |
| Do you understand that participation of your child is entirely voluntary? | Yes / No |
| Do you understand that your child is free to withdraw from this study  -at any time without any penalty?  -without having to give a reason?  -without affect your future health care (as applicable)?  Do you understand that your personal data collected in this Study shall be handled in accordance with Privacy Ordinance of Hong Kong? You acknowledge and agree that your records, data, and result of this Study will be reviewed by The Institutional Review Board of the University of Hong Kong/ Hospital Authority Hong Kong West Cluster and the Hong Kong Polytechnic University. You further understand that the results of this Study may be published in scientific journals, but you will not be identified in the publication.  ___________________________ ___________________________  Name of *Parent/ Guardian Signature of *Parent/ Guardian |  |
|  | Yes / No |
|  | Yes / No |
|  | Yes / No  Yes / No |
| 1. Assent of the child |  |
| Do you agree to participate in this study? | Yes / No |
| ___________________________ __________________________  Name of Child Signature of Child |  |

**Research Project Informed Assent Form**

**Project Title**

Combination Effect of Optical Defocus and Low Dose Atropine in Myopia Control – a Randomized Clinical Trial

**Research Team**

Dr. Ka Man CHUN, Prof. Chi Ho TO, Prof. Christopher Kai Shun LEUNG, Dr. Yan Yin TSE, Dr. Sheung Shun NG

We are doing a research study about the combination effect of optical defocus and low dose atropine on controlling the progression of shortsightedness.

If you decide that you want to be part of this study, you will be asked to wear the spectacle lenses and put on eye drops we provided (1 drop in the morning and 1 drop at night before bedtime) and we will do some measurement for your eyes.

There are some things about this study you should know. We will put eye drops to relax your eyes for examining the refractive power of your eyes. The eye drops will blur your vision at near for around 24-36 hours. And you may experience light sensitivity when you are at outdoor. These effects will ease off after 24-36 hours.

Not everyone who takes part in this study will benefit. A benefit means that something good happens to you. We think these benefits might be the control of the progression of shortsightedness.

When we are finished with this study we will write a report about what was learned. This report will not include your name or that you were in the study.

You do not have to be in this study if you do not want to be. If you decide to stop after we begin, that’s okay too. Your parents know about the study too.

If you decide you want to be in this study, please sign your name.

I, _________________________________, want to be in this research study.

___________________________________ ___________________

(Sign your name here) (Date)
